# The past, present, and future of sleep measurement in mild cognitive impairment and early dementia—towards a core outcome set: a scoping review

**DOI:** 10.1093/sleep/zsac077

**Published:** 2022-04-04

**Authors:** Jonathan Blackman, Hamish Duncan Morrison, Katherine Lloyd, Amy Gimson, Luke Vikram Banerjee, Sebastian Green, Rebecca Cousins, Sarah Rudd, Sam Harding, Elizabeth Coulthard

**Affiliations:** Bristol Medical School, University of Bristol, Bristol, UK; Bristol Brain Centre, North Bristol NHS Trust, Bristol, UK; Bristol Medical School, University of Bristol, Bristol, UK; Bristol Brain Centre, North Bristol NHS Trust, Bristol, UK; Bristol Medical School, University of Bristol, Bristol, UK; Bristol Brain Centre, North Bristol NHS Trust, Bristol, UK; Bristol Brain Centre, North Bristol NHS Trust, Bristol, UK; Bristol Brain Centre, North Bristol NHS Trust, Bristol, UK; Bristol Medical School, University of Bristol, Bristol, UK; Bristol Brain Centre, North Bristol NHS Trust, Bristol, UK; Bristol Brain Centre, North Bristol NHS Trust, Bristol, UK; Library and Knowledge Service, North Bristol NHS Trust, Bristol, UK; Research and Innovation, North Bristol NHS Trust, Bristol, UK; Bristol Medical School, University of Bristol, Bristol, UK; Bristol Brain Centre, North Bristol NHS Trust, Bristol, UK

**Keywords:** sleep, Alzheimer’s disease, mild cognitive impairment, MCI, AD

## Abstract

**Study Objectives:**

Sleep abnormalities emerge early in dementia and may accelerate cognitive decline. Their accurate characterization may facilitate earlier clinical identification of dementia and allow for assessment of sleep intervention efficacy. This scoping review determines how sleep is currently measured and reported in Mild Cognitive Impairment (MCI) and early dementia, as a basis for future core outcome alignment.

**Methods:**

This review follows the PRISMA Guidelines for Scoping Reviews. CINAHL, Embase, Medline, Psychinfo, and British Nursing Index databases were searched from inception—March 12, 2021. Included studies had participants diagnosed with MCI and early dementia and reported on sleep as a key objective/ outcome measure.

**Results:**

Nineteen thousand five hundred and ninety-six titles were returned following duplicate removal with 188 studies [*N*] included in final analysis. Sleep data was reported on 17 139 unique, diagnostically diverse participants (*n*). “Unspecified MCI” was the most common diagnosis amongst patients with MCI (*n* = 5003, 60.6%). Despite technological advances, sleep was measured most commonly by validated questionnaires (*n* = 12 586, *N* = 131). Fewer participants underwent polysomnography (PSG) (*n* = 3492, *N* = 88) and actigraphy (*n* = 3359, *N* = 38) with little adoption of non-PSG electroencephalograms (EEG) (*n* = 74, *N* = 3). Sleep outcome parameters were reported heterogeneously. 62/165 (37.6%) were described only once in the literature (33/60 (60%) in interventional studies). There was underrepresentation of circadian (*n* = 725, *N* = 25) and micro-architectural (*n* = 360, *N* = 12) sleep parameters.

**Conclusions:**

Alongside under-researched areas, there is a need for more detailed diagnostic characterization. Due to outcome heterogeneity, we advocate for international consensus on core sleep outcome parameters to support causal inference and comparison of therapeutic sleep interventions.

**Statement of Significance**

Sleep research in those with, or at risk of dementia is a topic of substantial interest through its possibility in providing early diagnostic biomarkers and disease modifying treatment targets. This scoping review uniquely determines how sleep is measured and reported in participants with early neurodegenerative disease thereby highlighting the past and present research landscape. We found outcome parameters, heterogeneously reported, a finding lending support for definition of a core sleep outcome set. The dominance of validated questionnaires despite technological advances and paucity of circadian and micro-architectural parameters are identified as future research opportunities. Finally, in light of large numbers of diagnostically undifferentiated participants we advocate for fuller characterization. Future adoption of these recommendations could accelerate progress in the field.

## Introduction

Sleep abnormalities and circadian rhythm disturbance are well recognized in established dementia [1–5]. These include objectively measured micro and macro-architectural changes, alongside subjective reports of reduced quantity and quality [2], all of which are disproportionately represented compared with age-matched controls and correlate closely with the severity of cognitive impairment [6,7]. Circadian rhythm disorders contributing to sleep disturbance are also generally more marked in those with dementia than in healthy aging [8]—possibly related to volumetric alterations in the Suprachiasmatic Nucleus influencing melatonin secretion [9].

As opposed to solely representing a marker of established disease, sleep abnormalities are increasingly recognized to occur much earlier in the natural history of dementia, during and even preceding the Mild Cognitive Impairment (MCI) stage [10,11]. Furthermore, many sleep disorders e.g. chronic insomnia, are associated with future increased risk of Alzheimer’s Disease (AD) dementia [12,13]. Rapid eye movement-sleep behavior disorder (RBD) is associated with future neurodegenerative synucleinopathies including Parkinson’s Disease (PD), Dementia with Lewy Bodies (DLB), and Multiple System Atrophy. In manifest PD, the presence of RBD is associated with future cognitive decline [14,15].

Whilst such abnormalities in sleep may reflect early symptomatic manifestation of pathology, there are also plausible mechanisms by which sleep abnormalities could precipitate or accelerate pathophysiological decline [16–20]. In AD, sleep abnormalities have been hypothesized to contribute to diminished clearance of a key pathognomonic feature—beta-amyloid [19,20], supported by work showing the unique role of Slow Wave Sleep (SWS) in removing intracerebral toxic breakdown products (including beta-amyloid) in mice [21]. Furthermore, SWS disruption in both healthy adults and those with AD is associated with greater levels of beta-amyloid pathology [22]. Similar mechanisms may be important in the α-synucleinopathy related dementias. For example, the co-occurrence of AD pathology is commonly seen in DLB [23,24] heralding more rapid cognitive decline [25,26] and is associated with the emergence of dementia in those with PD. Models of glymphatic clearance of toxic proteins remain similarly plausible for soluble phase α-synuclein but remain unproven.

Given that pathological changes associated with multiple subtypes of dementia predate symptomatic expression of symptoms by decades [27,28], a promising future strategy will be targeting early stages of the disease when pathology is more likely to be reversible and quality of life can be retained. Given that sleep abnormalities arise early, they may provide an ideal means to identify those at highest risk of dementia. In addition, optimizing sleep may delay progression of neurodegenerative disease whilst simultaneously promoting physiological processes that improve cognition (particularly long-term memory consolidation), general health, and wellbeing. As a result, there is much current interest in enhancing understanding of the precise nature of sleep abnormalities in dementia, their presence prior to onset of clinical symptoms, and trials of interventions to improve sleep disturbances.

However, whilst providing rich opportunity for deeper characterization and intervention, good sleep is a challenging concept to define, and therefore measure, in dementia—both due to its complex nature and the target population. As a multidimensional concept, sleep is measurable across levels and aspects [29,30]. For example, levels of measurement (measurement tools) may include self-report questionnaires, behavioral measures e.g. actigraphy, physiological means e.g. polysomnography, and less commonly analyses at the circuit or cellular level. Within each level, multiple aspects may be recorded (sleep parameters) e.g. sleep duration, efficiency, etc. Measuring sleep in MCI and early dementia is unique, encompassing challenges not seen in healthy populations [31], whilst also allowing for a wider range of techniques when compared to those in later-stage disease.

Improved characterization of sleep changes in dementia, assessing the relationship of changes with cognitive performance, and testing interventions to optimize sleep requires consistency in outcomes assessed and measures used. Recent systematic reviews into objective sleep measurement findings and sleep interventions in MCI were confined to narrative review due to outcome measure heterogeneity [11,32]. To our knowledge, there are no reviews describing current practices in measuring and reporting sleep in early dementia.

This paper presents the results of a scoping review designed to address two objectives. Firstly, to provide a report of the current landscape in early neurodegenerative sleep research by determining how sleep has been measured, the sleep parameters reported, and the means by which they are reported. Considering the growing interest between micro-architectural sleep parameters and the pathophysiology of early dementia [33–35], we gave particular focus to micro-architectural sleep in our manuscript. We identify areas with comprehensive data and highlight under-researched topics, providing also a description of how this is varying over time.

Our secondary objective is to determine the extent of heterogeneity in reported sleep measurement tools and parameters. Identifying this heterogeneity is important because it may preclude pooling and comparison of research on sleep in MCI or early dementia.

## Methods

### Protocol and registration

The original protocol for this scoping review was registered online with Figshare on 22^nd^ February 2021. A copy of the peer-reviewed and published protocol is available online (https://amrcopenresearch.org/articles/3-13). Minor amendments were made only where necessary to optimize the review process. In particular, the decision was taken not to date limit the search to allow for a full description of the research landscape over time.

### Eligibility criteria

#### Participants.

Each study must contain and provide sleep outcome measures for ≥ 1 subgroup consisting of participants meeting the following criteria:-

Inclusion criteria:

1. Adults aged greater than 18 (limit set to avoid excluding studies in genetic dementias); and
2. Male or Female; and

3. a) Satisfies established diagnostic criteria for MCI or has a clinical diagnosis of MCI;
  or
b) Satisfies established diagnostic criteria for dementia or would be expected to meet these criteria if study conducted before criteria established; and

4. At least 50% of reported data in participants with mild severity disease as evidenced by: MMSE ≥ 20 or CDR < 2 or an equivalent measure.

Exclusion criteria:

1. Studies reporting only on groups with a diagnostic a mix of participants e.g. with and without a diagnosis of dementia.

#### Concept.

All included studies met the following two concept criteria:-

1. Sleep measurement/assessment is a key component of interest as evidenced by one or more objective relating to sleep defined within the original aims and objectives of the study;

and

2. Sleep outcomes/parameters e.g. total sleep time, sleep efficiency, subjective experience of sleep are reported through use of validated sleep outcome measure/tool.

#### Context.

Studies were conducted in either or both community and health-care settings.

#### Types of evidence sources.

All published, peer-reviewed articles written in English, specifically those reporting both experimental and quasi-experimental study designs including randomized controlled trials, before and after studies, and interrupted time-series studies. In addition, analytical observational studies including prospective and retrospective cohort studies, case-control studies, and analytical cross-sectional studies as well as descriptive observational study designs including case series and descriptive cross-sectional studies were considered for inclusion alongside qualitative studies. Review papers, individual case reports, text, and opinion papers were excluded.

This scoping review was conducted in accordance with the Joanna Briggs Institute (JBI) methodology for scoping reviews [36].

### Information sources

To identify potentially relevant studies, a literature search of CINAHL, Embase, Medline, Psychinfo and British Nursing Index databases were searched from inception to the present day (12/03/2021). The search strategies were drafted by an experienced clinical librarian (SR) and further refined by team consensus.

### Search

The full electronic search strategy, is available in Supplementary Material 1.

### Selection of sources of evidence

Following the search, all identified citations were collated and uploaded into reference management software (Endnote) with duplicates removed automatically. Titles of studies clearly unrelated to the participants and concept of the scoping review were removed. Two senior reviewers (JB and HM) independently reviewed 10% of the remaining abstracts against the inclusion criteria as stated with agreement above 90%. Remaining abstracts were then distributed equally among the six members of the reviewing team (JB, HM, KL, AG, SG, RC) with 10% of all allocated abstracts reviewed by JB or HM to ensure agreement was above 90%. All full-texts were screened independently by two reviewers (any two of JB, HM, SH, SG, KL, AG, LB, RC) with regular consensus meetings. Reasons for exclusion of sources at full-text stage were recorded. Discrepancies were resolved by team consensus.

Nineteen thousand five hundred and ninety-six titles were returned following duplicate removal. Nine hundred and thirty-eight articles were selected for full-text review. Seven hundred and fifty articles were excluded, most commonly as the article was not a peer-reviewed full text e.g. conference abstract (*n* = 396) and due to overly advanced dementia severity (*n* = 190) leaving 188 studies included in the final analysis (for full reference list see Supplementary Material 3).

**Figure 1.**
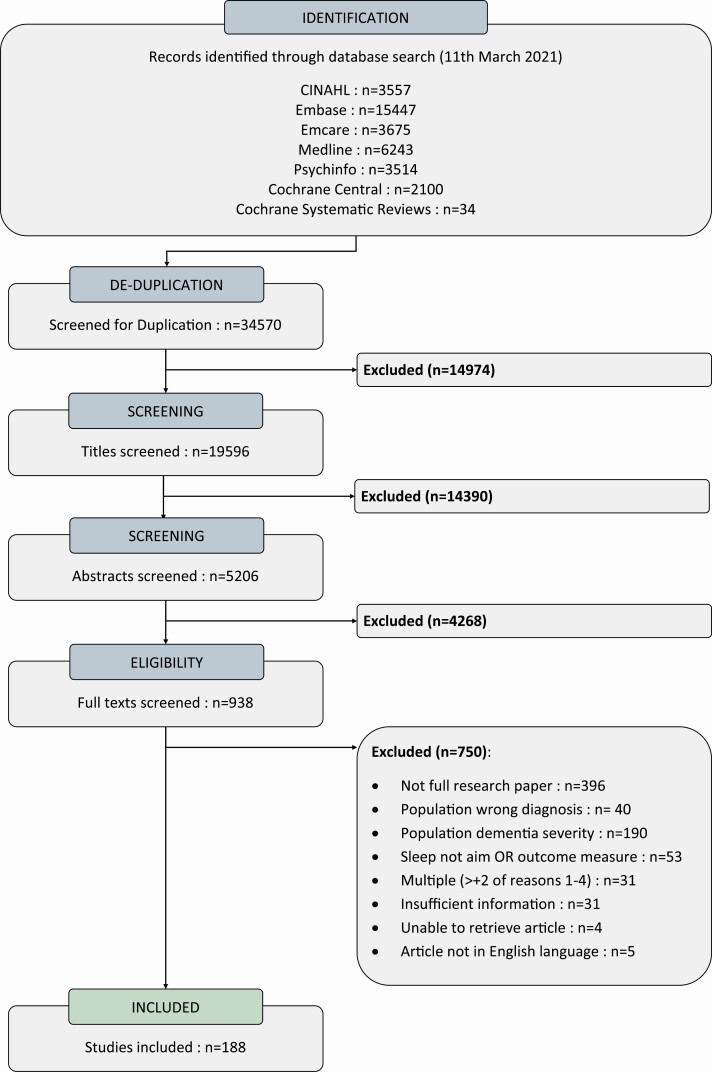
Prisma-Scr flow diagram. Abbreviations: CINAHL, Cumulative Index to Nursing and Allied Health Literature.

The literature search and article selection process is reported as per the Preferred Reporting Items for Systematic Reviews and Meta-Analyses extension for scoping review (PRISMA-ScR) flow diagram [37] (see Figure 1).

### Data charting process

Data were extracted from included articles by a member of the reviewing team (JB, HM, AG, KL, LB) onto a live shared database. Data quality was checked by senior reviewers for completeness and record consistency.

### Data items

Data extracted included details of the participants (number, age, gender, cognitive scores, diagnosis), study (date, type, location, and setting), validated outcome measures (sleep measurement tools), and sleep parameters reported from each tool. For a complete description of data, fields collected see Table 1. In the case of missing, unclear, or incomplete data, attempts were made to contact authors. As a scoping review, this work was designed to determine the nature of sleep outcomes reported rather than evaluate study quality, as such critical appraisal and risk of bias analysis were not undertaken.

**Table 1.** Data extraction template

	Data field	Options	Explanation
Study information	Authors		
	Title		
	Year		
	Journal		
	Country		
	Participant Type	*In-Patient* *Out-Patient*	To determine study setting i.e. community vs healthcare.
	Possible Duplicate Study Population	*Yes* *No*	To identify instances where multiple papers are reporting on the same study/ study population
	Sleep Measurement Location	*Sleep Laboratory* *Hospital* *Home* *Multiple (inc Sleep Lab)* *Multiple (exc Sleep Lab)*	
	Study Type	*Observational* *Interventional* *Validation*	
Overall participants	Total Participant Number		
	Number Female		
	Mean Age		
For each subgroup	Participant Number		Data recorded for each eligible subgroup within each paper
	Mean age		
	Number Female		
	MCI/ dementia	*MCI* *Dementia*	
	MCI/ dementia type		
For all measurement tools	Measurement tool name		To identify all measurement tools used to produce sleep outcome metrics
	Number of nights recorded		
For all sleep parameters	Name		Each reported sleep parameter recorded and its means of measurement
	Measurement Tool utilized		

Adapted from [36].

### Synthesis of results

R Studio v1.4.1 “Tiger Daylily” software was used for data cleaning, analysis, and figure production.

## Results

### Characterization of sources of evidence

One hundred and eighty-eight included full-texts presented data on 18 770 participants (n) and drew data from 178 unique studies (N) involving 17 139 unique participants, mean age 73.7, female gender 55.4%. Studies were published between 1982 and 2021 (see Table 2).

**Table 2.** Included study and participant characteristics

	Paper number (*N*)	Study percentage	Participants (*n*)	Participant percentage/ average	Missing data
Total included	188		18 770		
Total unique	178		17 139		
Female gender			8101	55.4	*N* = 17, *n* = 2515
Mean age			16 130	73.71	*N* = 13, *n* = 1009
Study type					
*Interventional*	28	14.89	1231	7.18	
*Observational*	158	84.04	15 783	92.09	
*Validation*	2	1.06	125	0.73	
Study population					
*In-patient*	1	0.53	101	0.59	
*Out-patient*	184	97.87	16 824	98.16	
*Both*	2	1.06	73	0.43	
*Unspecified*	1	0.53	141	0.82	
Cognitive measures					
*MMSE*	126	67.02	10 987	26.26	
*MOCA*	20	10.64	1465	23.98	
*CDR*	25	13.3	2041	1.31	
*ADAS-COG*	8	4.26	804	31.9	
*ACE*	2	1.06	74	81.2	
*GDS*	3	1.6	106	3.8	

Participants had a wide range of often incompletely delineated diagnoses with “MCI of an Unspecified Etiology” comprising the largest proportion of patients with MCI (*n* = 5003, 60.6% total MCI) with more specific diagnoses uncommon e.g. AD-MCI (*n* = 293, 3.6% total MCI). Of 8894 participants with early dementia, the majority had a diagnosis of AD (*n* = 7563, 85% total dementia—see Supplementary Figure 1).

### Sleep measurement tools

Sleep was measured most commonly by a range of validated questionnaires/ diaries (participants[*n*] = 12 586, studies[*N*] = 131). By participant number these included most commonly the Pittsburgh Sleep Quality Index (PSQI) [*n* = 5786, *N* = 58], the Epworth Sleepiness Scale (ESS) [*n* = 3018, *N* = 49], the Neuropsychiatric Inventory (NPI) [*n* = 1598, *N* = 15] measure of sleep disturbance, the REM Sleep Behavioral Disorder Screening Questionnaire (RBD-SQ) [*n* = 701, *N* = 7], the Mayo Sleep Questionnaire (MSQ) [*n* = 579, *N* = 6], the Consensus Sleep Diary [*n* = 461, *N* = 12] and the Insomnia Severity Index (ISI) [*n* = 396, *N* = 8]. The Clinical Global Impression of Change (CGI-C) was used in a large number of participants but only one study [*n* = 3800, *N* = 1]. For the full list of see Supplementary Material 3.

**Figure 2.**
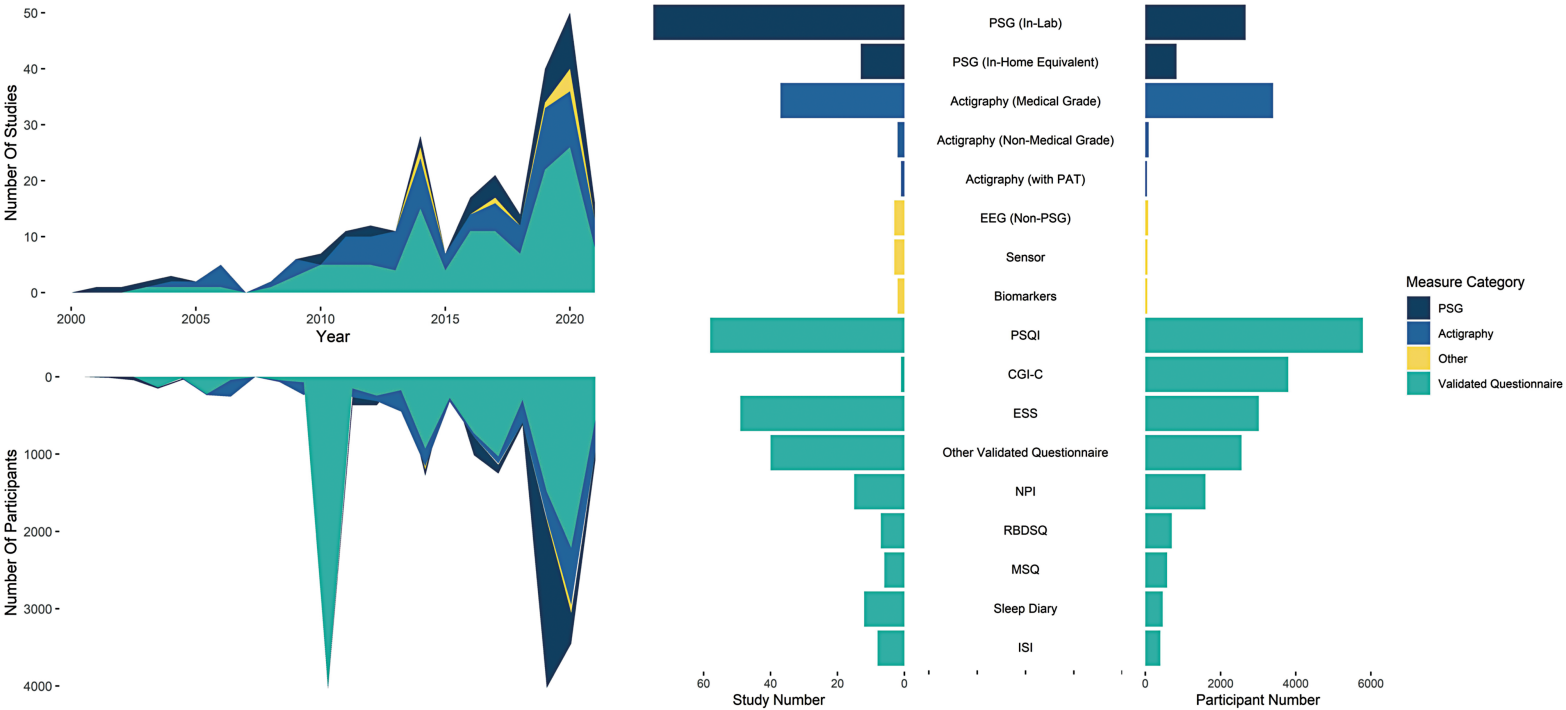
Utilized measurement tools. (a) Relative proportion of participants and studies utilizing PSG, actigraphy, validated questionnaires, and other means to report sleep parameters over time. (b) Overall representation of PSG, actigraphy, validated questionnaire, and other means to report sleep described in the literature by number of studies and participants. Abbreviations: LBD, Lewy Body Disease—encompassing Parkinson’s Disease Dementia and Dementia with Lewy Bodies; PAT, Peripheral Arterial Tomography; PSQI, Pittsburgh Sleep Quality Index; CGI-C, Clinical Global Impression of Change; ESS, Epworth Sleepiness Scale; NPI, Neuropsychiatry Index; RBDSQ, REM Sleep Behavioural Disorder Screening Questionnaire; MSQ, Mayo Sleep Questionnaire; ISI, Insomnia Severity Index.

The proportion of participants undergoing sleep measurement utilizing each measurement tool has changed minimally over the last two decades despite an overall clear increase in interest within the area (see Figure 2a).

Fewer participants underwent polysomnography (PSG) (n = 3492, N = 88) and medical grade actigraphy (n = 3395, N = 37), with minimal adoption of non-PSG Electroencephalograms (EEG) (n = 74, N = 3) (see Figure 2b)

### Sleep parameters in MCI and early dementia

**Figure 3.**
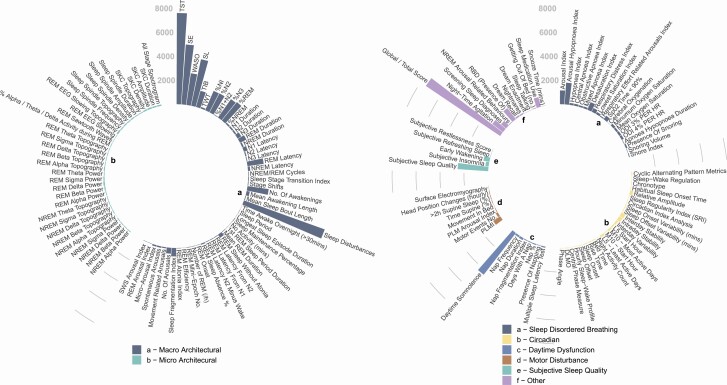
Reported sleep parameters. (a) Number of participants with reported data on each macro and micro-architectural parameter. (b) Number of participants with reported data on remaining outcome parameters split into sleep-disordered breathing, circadian rhythm, daytime dysfunction, motor disturbance, subjective sleep quality, and “other” categories. Abbreviations: REM, Rapid Eye Movement; NREM, Nonrapid Eye Movement; ODI, Oxygen Desaturation Index; PLM, Periodic Limb Movement; PLMI, Periodic Limb Movement Index; DLMO, Dim Light Melatonin Onset.

After synonymous parameters were combined, a total of 165 separate sleep parameters were reported across all studies, which were divided by theme into eight categories, macro-architectural, micro-architectural, sleep-disordered breathing, motor activity, daytime metrics, circadian rhythm, subjective sleep quality and “other” (see Figure 3). As shown, metrics relating to macro-architecture, sleep-disordered breathing, subjective experience of sleep, and daytime disturbance are relatively well represented whilst those pertaining to micro-architecture, circadian rhythm, and motor disturbance are considerably less well reported. Within the motor disturbance category, whilst 730 participants had the Periodic Limb Movement Index (PLMI) reported, the remaining 7 parameters were sparsely reported. Circadian rhythm parameters were reported in a total of 364 participants across 25 separate studies. Furthermore, 25 separate parameters were reported with only relative amplitude, interday stability, interday variability, L5 least active days, M10 most active days reported in over 100 participants.

This heterogeneity in outcome parameters was mirrored across the dataset. 62/165 (37.6%) total parameters were described only once in the literature and over half—97/165 (58.8%) were reported in at most two studies.

Of the 25 most commonly reported sleep parameters, as expected, by study, total sleep time (TST) was reported most frequently (*N* = 111), followed by sleep efficiency (SE) (*N* = 102) and sleep latency (SL) (*N* = 72) (see Supplementary Figure 2). Total scores of validated questionnaires were also reported frequently (*N* = 108). Commonly reported macro-architectural measures were reported in approximately equal numbers across PSG, actigraphy, and questionnaires.

### Assessment of insomnia

As a key prominent subjective sleep disturbance [38] we also specifically assessed reporting of insomnia. *N* = 16 studies reported total scores from validated questionnaires to assess insomnia or directly reported subjective insomnia. Whilst 9/16 included an Epworth Sleepiness Scale or equivalent to assess daytime function, none included sleep diary data in contrast to proposed reporting standards [39].

### Micro-architectural sleep parameters

Only 360 participants in total with diagnoses spanning the spectrum from MCI to early dementia have had micro-architectural sleep parameters reported across 12 separate studies. Of the 34 individual sleep parameters reported, 13 (38.2%) are reported by a maximum of 1 study (see Table 3).

**Table 3.**
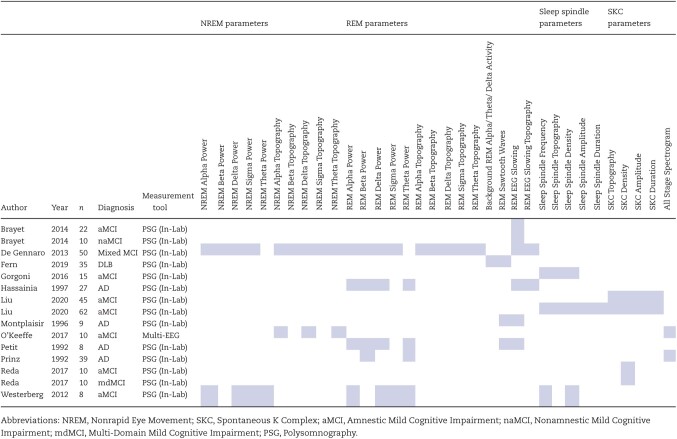
Micro-architectural parameters reported by study

Of note, despite a broad range of reported micro-architectural parameters described, no included study reported on specific parameters more recently associated with overnight memory consolidation for example, R slow-wave activity differentiated by faster (1–4 Hz) and slower (<1 Hz) frequencies or on sleep spindle/ slow oscillatory synchrony [40].

### Sleep outcome reporting in interventional studies

Twenty-eight studies evaluated interventions hypothesized to influence sleep. Amongst these studies, 55 separate parameters were described. However, there was substantial heterogeneity in reporting with direct comparison possible only infrequently. This was possible for Total PSQI Score (*N* = 8/23), SE by actigraphy and PSG (*N* = 7/23), Total Epworth Sleepiness Score (*N* = 6/23), and TST by actigraphy and PSG (*N* = 6/23). SE by any measurement tool was reported in 57.1% of studies, TST in 53.6%, and SL in 50%. These latter measures were also presented in approximately equal proportions of validated questionnaires, actigraphy, and polysomnography. Of the remaining parameters, 22 were reported in only one study (see Supplementary Figure 3).

## Discussion

Overall there was a rich diversity in both the means by which sleep has been measured and the outcome parameters reported in a population with MCI and early dementia. However, in exploring this overall landscape, several themes emerge.

Firstly, whilst large numbers of participants with MCI and early dementia have been involved in sleep research and those with early dementia typically had an underlying cause stated, the underlying pathophysiological diagnosis in those with MCI was often unclear or unspecified. MCI itself is recognized as a heterogeneous group of disorders reflecting an “at-risk” state for the development of dementia [41]. Even when participants are selected as part of rigorous adherence to established diagnostic criteria, underlying pathophysiology is likely to vary considerably [42]. Indeed after the removal of studies comprising unspecified MCI or comprising of groups of mixed causes, the number of studies assessing sleep in well-characterized/delineated MCI diminishes substantially comprising only 12.1% of participants with MCI. This is highly likely to be due in part to inherent difficulties in identifying this cohort with the need for neuroimaging and fluid biomarkers and establishing this diagnosis prior to potential progression to early dementia. Nonetheless, we advocate that it is in these well-delineated groups where valuable mechanistic insights probing links between sleep and neurodegeneration may be found. A future in which aspects of disordered sleep may be used as a further biomarker for early identification of specific neuropathological change demands that pathology is first matched to a sleep abnormality or characteristic, a task rendered far more challenging in diagnostically diverse or uncharacterized populations. As a pragmatic step, where possible, we advocate for avoidance of aggregate reporting of sleep data on mixed MCI populations. We suggest at a minimum study groups should consist of clinically homogenous MCI participants. Ideally, MCI groups would be classified according to the most up-to-date biomarkers into biologically specific categories (e.g. MCI due to AD), however, very few patients receive a biomarker diagnosis outside research. Therefore for studies looking to maximize cohort size and potential statistical inference, combining biomarker-driven cohorts with clinically comparable groups could be considered e.g. studies reporting on AD-MCI could plausibly include amnestic MCI but exclude nonamnestic participants.

Secondly, a majority of studies reported sleep outcomes utilizing only validated questionnaires. These have multiple advantages including providing insights into the subjective experience of sleep, ease of administration, and low participant burden [43]. Indeed, even when used as part of interventional studies, improving quality of life and reducing symptomatic sleep disturbance is clearly important and may be the sole objective of a study. However, many sleep parameters can be measured only by objective means and there is known to be discrepancy in subjective vs. objective sleep disturbance in MCI and early dementia [44,45]. These factors together reduce the power of any study relying solely on questionnaires to detect abnormalities specific to MCI/ early dementia or to assess intervention efficacy, particularly those which could plausibly alter disease progression through e.g. SWS optimization [19,46].

Thirdly, and in contrast to the above, commonly reported symptomatic sleep disturbances such as insomnia are less well characterized by objective measures [47]. Indeed, guidelines suggest that optimal evaluation and differential diagnosis of insomnia should be achieved through self-administered questionnaires and be supplemented by a minimum of a two-week sleep diary together with a measure of daytime dysfunction [39]. Here, amongst studies reporting insomnia, we found that none included sleep diary data and only 9/16 described daytime dysfunction, suggesting that within the literature, diagnosis, and quantification of this important symptom related to quality of life could be optimized.

Fourthly, of note, is the relative lack of adoption of home-based non-PSG EEG recording devices having been used in only 74 participants in 3 separate studies. Such devices have the advantage of capturing single or multi-channel EEG metrics, desirable for further interrogation of the relationship between cognitive performance and sleep and providing micro-architectural information specifically linked to brain health [48], but within the home environment. This allows for longer durations of sleep monitoring and minimization of both first-night effects and observational bias when compared to in-laboratory recordings [49]. Such devices also have emerging favorable evidence of validation with single-channel devices, for example, capable of delineating sleep stages, specifically in assessing REM duration, combined N2/N3 duration, and also frontal slow-wave activity [50]. Multi-channel devices have been shown capable of acquiring signals comparable to PSG and also produce automated sleep staging with similar performance to expert rating of PSG data [51]. In-laboratory assessments are also uniquely challenging in groups with cognitive impairment due to the risk of disorientation and increased participant discomfort. Of recent relevance, they are also less susceptible to interruption of availability in the context of the COVID-19 Pandemic. Reasons for delayed adoption may include a lack of validatory studies in older adults and those with cognitive impairment. Nonetheless whilst PSG is a gold-standard in terms of recording quality, due to these limitations, the increasing market availability of wearable, home-based EEG devices may offer significant advantages, particularly in this population.

Fifthly, there was a relative paucity of information regarding circadian rhythmicity, motor disturbance and micro-architectural measures of sleep. Multiple facets of sleep micro-architecture have been linked with aging [33], cognitive performance [34,35], and pathological features of dementia [19,52]. For example, episodic memory consolidation is proposed to be enhanced during NREM due to the combined activity of slow oscillations (<1Hz), hippocampal ripples, and thalamocortical sleep spindles [53]. Deposition of amyloid-beta is similarly associated with reduced slow oscillatory (<1 Hz) activity as opposed to age-related changes seen throughout the remaining frequency range (1–4 Hz) [54,55]. These associations have been found in rodent models [56] and in presymptomatic individuals but are seemingly untested in an early symptomatic AD group representing a missing translational step in understanding.

Aging has been associated with progressive disturbance in circadian rhythm [57] and there is correlation between cognitive performance and chronotype with e.g. phase advance associated with lower cognitive scores [58] and also predicting incident dementia [59]. Nonetheless, replication of these findings amongst an early, MCI population is not found in significant numbers within this dataset with the most commonly described circadian parameter “interday stability” described in only 364 participants. Consolidation and comparison of such findings is hindered by the use of a wide breadth of synonymous terminology which, even after concatenation into parameters reflecting individual characteristics, left 25 separate parameters pertaining to circadian rhythm reported.

Finally, given the suspected bidirectionality in sleep disturbance and cognition, results of interventional studies to delay onset or progression of symptoms need to be compared. At present, however, outcomes are reported highly heterogeneously inhibiting comparison and synthesis/ pooling of data. In studies testing interventions to optimize sleep, it was surprising to find that only approximately 60% reported basic metrics such as TST. Many outcomes were reported only in one study, which whilst again adding breadth is subject to issues including the risk of positive-finding publication bias. Further, the means of measurement and reporting was diverse, with approximate equal use of questionnaires, actigraphy, and PSG. Questionnaires, whilst individually validated, are not necessarily validated against each other and given the risk of recall bias and subjective/objective discrepancy particularly prevalent in this population should perhaps be interpreted with caution. Most interventions are unlikely to be without side-effect and therefore side-by-side comparison of efficacy would be highly desirable in order to inform optimal clinical choice of intervention in the future. For these reasons, particularly in interventional studies, we advocate for a core (but expandable) outcome set comprised of parameters described with consistent terminology. Given the small numbers of participants with defined neuropathological causes of MCI, such an outcome set would also be of use in observational studies in order to consolidate and reproduce findings with more confidence. While we acknowledge the diverse range of sleep targets e.g. sleep apnea and insomnia where different primary outcomes may be relevant, we propose that there are core sleep metrics that should be reported across all studies in addition to core outcome of interest—for example, one would not look at desaturation alone while paying no attention to sleep duration and vice versa. For an example of candidate core sleep metrics, see Table 4. Beyond advocating for the use of validated questionnaires as a minimum, we suggest that prescribing specific objective measurement tools would be unhelpful. However if such measurement tools e.g. actigraphy/PSG are used, based on the results from this review, we propose that adherence to reporting the modest set of parameters highlighted for each in Table 4 would substantially boost comparison between studies. This list of metrics is not intended to be exhaustive, but provided as a basic format including commonly reported metrics but also those of particular significance for brain health/ dementia from which we hope a core outcome set could evolve in the future, determined through international consensus and informed by data-driven studies [48].

**Table 4.**
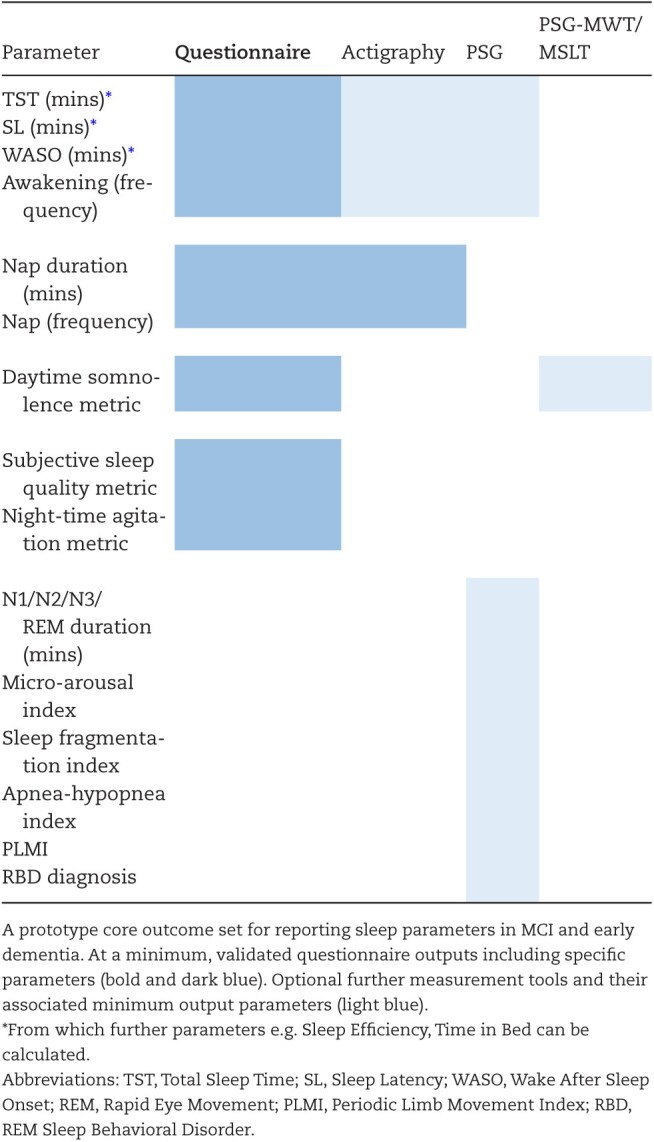
Prototype core outcome set

This review is subject to several limitations. In terms of article selection, we opted to include only articles whose starting population included at least one subgroup of individuals with MCI or early dementia. We, therefore, excluded longitudinal studies whose purpose may have been to determine incident risk of dementia from various exposure variables including sleep. This was due to our focus on measuring sleep within diagnosed MCI/early dementia as opposed to healthy individuals who may or may not progress to dementia. We opted to include studies comprised of a majority of individuals with mild severity of dementia but could plausibly include those with moderate or severe dementia. We felt, on balance, that including these studies increases the overall representation of sleep research in early dementia acknowledging that some studies are not diagnostically homogeneous in this way. Whilst we have recorded the methods and outcomes reported, as this is a scoping review following JBI guidelines we do not evaluate study quality or undertake risk of bias analyses. We sought to identify individual papers which referred to separate findings from the same study and recorded this information when discovered. However, such information is not always readily available, and it is possible that our estimate of unique participants may contain inaccuracies.

**Box 1—Summary of recommendations for early neurodegenerative sleep research**

- International consensus on a core (but expandable) outcome set of sleep parameters
- Where possible, characterization of underlying pathophysiology of MCI
- Use of objective means of sleep measurement in concert with validated questionnaires
- Consideration of use of noninvasive, home-based non-PSG EEG devices
- Where possible further assessment of micro-architectural and circadian metrics which are relatively under-reported

## Conclusion

There is a rich diversity of sleep outcome measures reported in MCI and early dementia, however, this heterogeneity inhibits comparison across studies and clinical groups. Furthermore, sleep is reported in relatively diagnostically undifferentiated cohorts and means of measuring sleep have remained static despite technological advances. Alongside identifying under-researched areas and relative undercharacterization of MCI populations, here we advocate for international consensus on a core set of sleep outcome measures to enable causal inference and direct comparison of therapeutic sleep interventions in this patient cohort.

## Supplementary Material

zsac077_suppl_Supplementary_Figure_S1

zsac077_suppl_Supplementary_Figure_S2

zsac077_suppl_Supplementary_Figure_S3

zsac077_suppl_Supplementary_Material

## Data Availability

The data underlying this article will be shared on reasonable request to the corresponding author.
