# Supplementary figures and images for: The past, present, and future of sleep measurement in mild cognitive impairment and early dementia—towards a core outcome set: a scoping review
Source: Sleep. 2022 Apr 4;45(7):zsac077. doi: 10.1093/sleep/zsac077 (PMC9272273; doi:10.1093/sleep/zsac077)

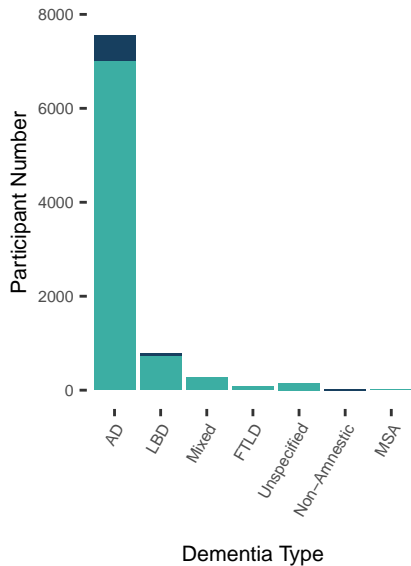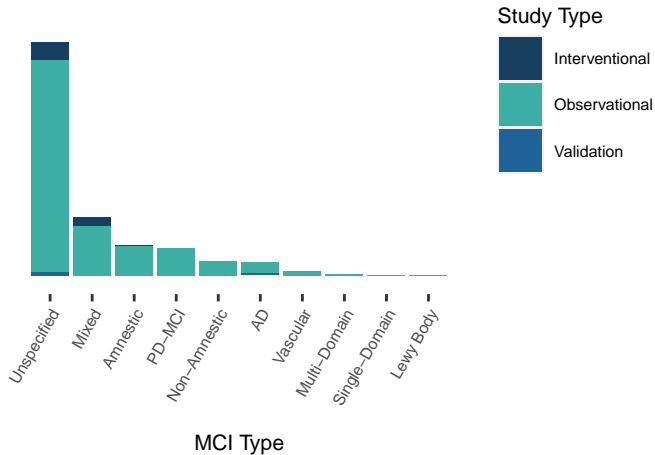

Supplement: zsac077_suppl_Supplementary_Figure_S1 [file zsac077_suppl_supplementary_figure_s1.pdf]

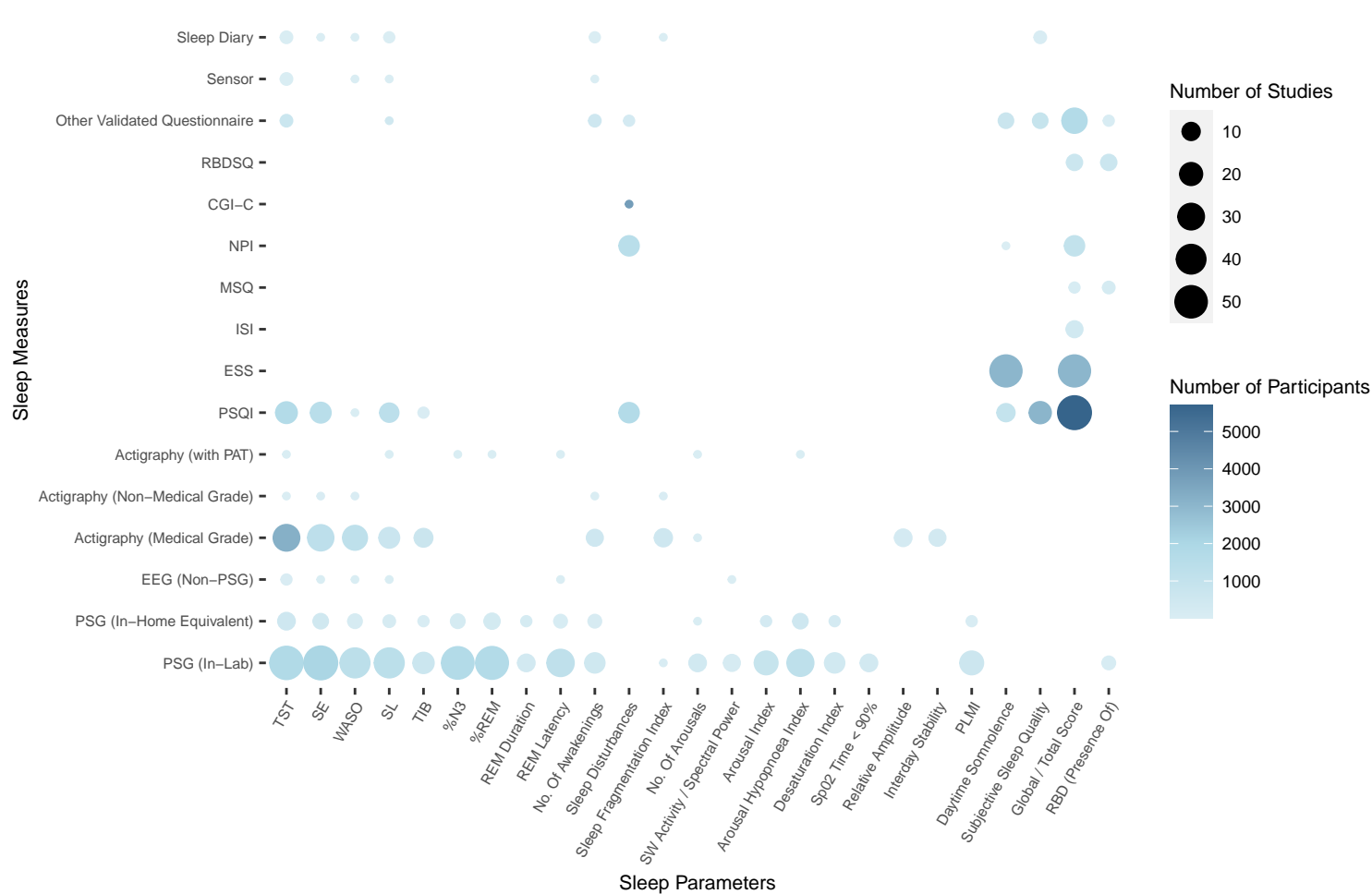

Supplement: zsac077_suppl_Supplementary_Figure_S2 [file zsac077_suppl_supplementary_figure_s2.pdf]

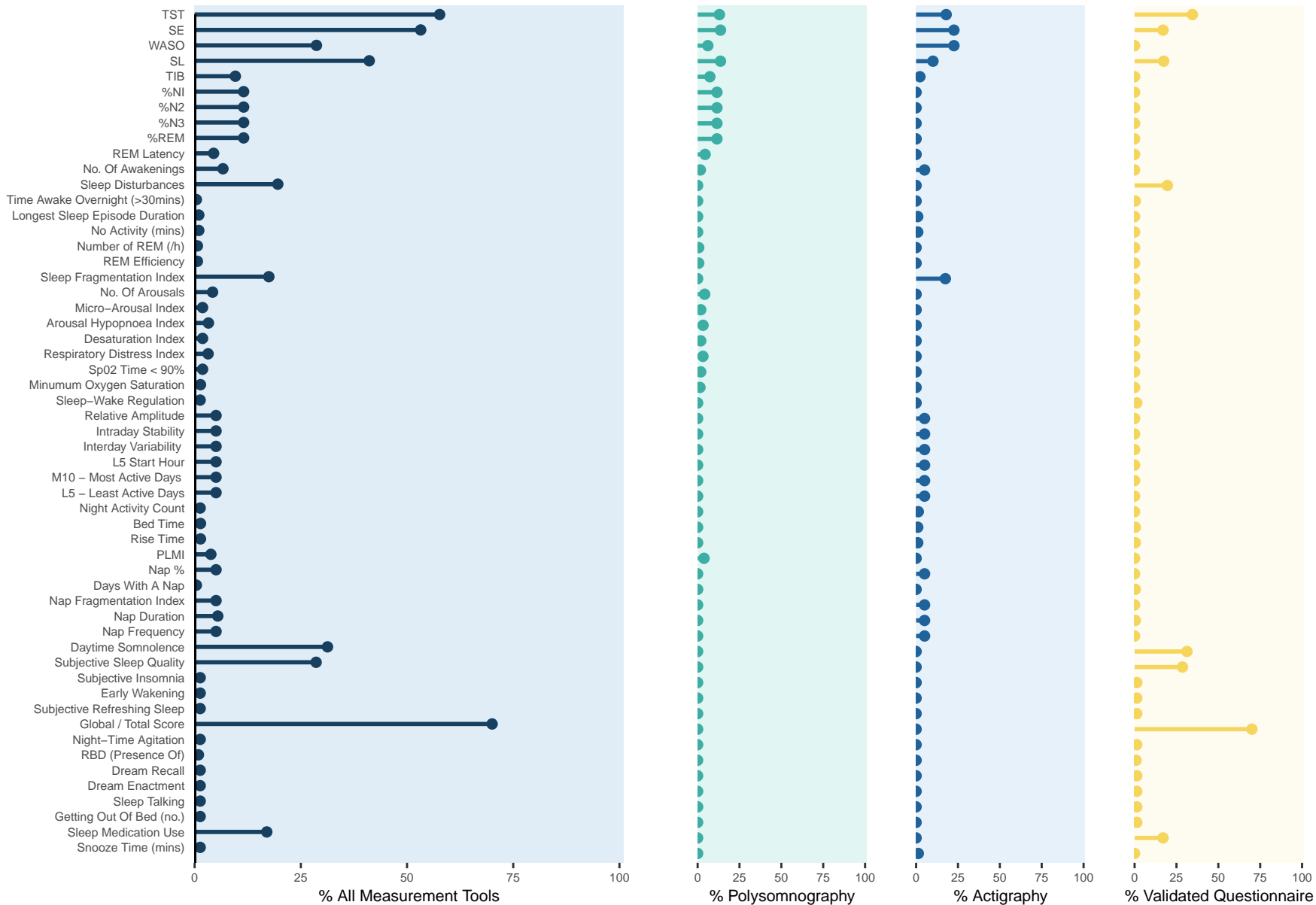

Supplement: zsac077_suppl_Supplementary_Figure_S3 [file zsac077_suppl_supplementary_figure_s3.pdf]
