# Supplementary material for: The past, present, and future of sleep measurement in mild cognitive impairment and early dementia—towards a core outcome set: a scoping review
Source: Sleep. 2022 Apr 4;45(7):zsac077. doi: 10.1093/sleep/zsac077 (PMC9272273; doi:10.1093/sleep/zsac077)
Supplement: zsac077_suppl_Supplementary_Material [file zsac077_suppl_supplementary_material.docx]

**The Past, Present and Future of Sleep Measurement in Mild Cognitive Impairment and Early Dementia – Towards a Core Outcome Set: A Scoping Review**

Supplementary Materials

Jonathan Blackman^1,2*^(jonathan.blackman@bristol.ac.uk)

Hamish Duncan Morrison^1,2*^ (hamish.morrison@bristol.ac.uk)

Katherine Lloyd^1,2^

Amy Gimson^2^

Luke Vikram Banerjee^2^

Sebastian Green^1,2^

Rebecca Cousins^2^

Sarah Rudd^3^

Sam Harding^4^

Elizabeth Coulthard^1,2^ ([elizabeth.coulthard@bristol.ac.uk](mailto:elizabeth.coulthard@bristol.ac.uk))

*Joint first authors

Affiliations : 1. Bristol Medical School, University of Bristol, Bristol, BS2 8DZ, UK

2. Bristol Brain Centre, North Bristol NHS Trust, Bristol, BS10 5NB, UK

3. Library and Knowledge Service, North Bristol NHS Trust, Bristol, BS10 5NB, UK

4. Bristol Brain Centre, North Bristol NHS Trust, Bristol, BS10 5NB, UK

Corresponding Author : Dr Jonathan Blackman

Institute of Clinical Neurosciences, Bristol Medical School

Learning & Research Building, Southmead Hospital

University of Bristol, Bristol, BS10 5NB, UK.

Section 1 – Search Strategy

Database: Embase <1974 to 2021 March 11> , Ovid Emcare <1995 to 2021 Week 08>, Ovid MEDLINE(R) ALL <1946 to March 11, 2021>

Search Strategy:

--------------------------------------------------------------------------------

1 (Sleep* or slept or insomni*).ti,ab. (577549)

2 Sleep/ or exp insomnia/ (271166)

3 1 or 2 (644918)

4 (MCI or MNCD or AAMI or AACD or MCD or ARCD or NMCI or AMCI or MMCI or SMCI or MCIA or LBD or FTLD or FTD).ti,ab. (102981)

5 exp Dementia/ (654822)

6 (dement* or alzheim* or lewy or frontotemporal).ti,ab. (654677)

7 ((Cognit* or Neurocognit* or Neurodegenerat*) adj3 (disorder* or impair* or declin* or dement* or deficit* or dysfunction* or disease* or impair*)).ti,ab. (621228)

8 4 or 5 or 6 or 7 (1252198)

9 3 and 8 (39233)

10 exp animals/ not exp humans/ (9905564)

11 (measur* or assess* or question* or evaluat* or quanti* or apprais* or guage* or guaging or test* or examin* or inventor* or survey*).ti,ab. (31799186)

12 9 and 11 (26051)

13 12 not 10 (24725)

14 13 (24725)

15 limit 14 to english language (23559)

16 15 use medall (6243)

17 exp sleep/ (393012)

18 exp insomnia/ (105371)

19 1 or 17 or 18 (733476)

20 exp dementia/ (654822)

21 4 or 6 or 7 or 20 (1252198)

22 exp animal/ not exp human/ (9905564)

23 11 and 19 and 21 (27974)

24 23 not 22 (26622)

25 24 (26622)

26 limit 25 to english language (25396)

27 26 use oemezd (15447)

28 26 use emcr (3675)

29 15 or 27 or 28 (25365)

Section 2 – Supplementary Figures

Supplementary Figure 1 Title

**SM Figure 1** – Included Population by Diagnosis

Supplementary Figure 1 Caption

(a) Number of included participants with dementia split by underlying diagnosis. (b) Number of included participants with MCI split by underlying cause.

Abbreviations : AD (Alzheimer’s Disease), DLB (Dementia with Lewy Bodies), FTLD (Fronto-Temporal Lobe Dementia), MSA (Multiple System Atrophy), PD-MCI (Parkinson’s Disease Mild Cognitive Impairment)

Supplementary Figure 2 Title

**SM Figure 2** – Commonly reported sleep parameters by measurement tool, participant number and study number

Figure 2 Caption

Abbreviations : TST (Total Sleep Time), SE (Sleep Efficiency), WASO (Wake After Sleep Onset), SL (Sleep Latency), TIB (Time in Bed), REM (Rapid Eye Movement), SW (Slow Wave), PLMI (Periodic Limb Movement Index), RBD (REM Sleep Behavioural Disorder)

Supplementary Figure 3 Title

**SM Figure 3** – Sleep Parameters Reported in Interventional Studies

Figure 3 Caption

Footnote : (a) Proportion of total interventional studies expressing each sleep parameter (b) Proportion of total interventional studies expressing each sleep parameter measured by polysomnography, actigraphy (c) and validated questionnaire (d).

Abbreviations : TST (Total Sleep Time), SE (Sleep Efficiency), WASO (Wake After Sleep Onset), SL (Sleep Latency), TIB (Time In Bed), REM (Rapid Eye Movement), PLMI (Periodic Limb Movement Index), RBD (REM Sleep Behavioural Disorder)

Section 3 – Included Papers

1. An C, Yu L, Wang L, Jin G, Song M, Zhu Q, et al. Association between sleep characteristics and mild cognitive impairment in elderly people. Neurophysiology. 2014;46(1):88-94.

2. Bademli K, Lok N, Canbaz M, Lok S. Effects of Physical Activity Program on cognitive function and sleep quality in elderly with mild cognitive impairment: a randomized controlled trial. Perspectives in psychiatric care. 2019;55(3):401‐8.

3. Bahk WM, Song HR, Woo YS, Wang HR, Jun TY, Kim MD, et al. Effect of the timing of acetylcholinesterase inhibitor ingestion on sleep. International journal of neuropsychopharmacology. 2014;17:70.

4. Bahnasy WS, El-Heneedy YAE, Ragab OAA, Badr MY, Seleem MAH, Amer RAR, et al. Polysomnography, brain volumetry, and mismatch negativity as early biomarkers of amnestic mild cognitive impairment progression. Egyptian Journal of Neurology, Psychiatry and Neurosurgery. 2018;54(1):20.

5. Basta M, Simos P, Vgontzas A, Koutentaki E, Tziraki S, Zaganas I, et al. Associations between sleep duration and cognitive impairment in mild cognitive impairment. Journal of Sleep Research. 2019;28(6):e12864.

6. Basta M, Zaganas I, Simos P, Koutentaki E, Dimovasili C, Mathioudakis L, et al. Apolipoprotein E ɛ4 (APOE ɛ4) Allele is Associated with Long Sleep Duration Among Elderly with Cognitive Impairment. Journal of Alzheimer's Disease. 2021;79(2):763-71.

7. Bliwise DL, Keating GL, Rye DB, Hu WT, Trotti LM. Cerebrospinal Fluid Hypocretin and Nightmares in Dementia Syndromes. Dementia and Geriatric Cognitive Disorders Extra. 2021:19-25.

8. Bliwise DL, Scullin MK, Trotti LM. Fluctuations in cognition and alertness vary independently in dementia with Lewy bodies. Movement Disorders. 2014;29(1):83-9.

9. Bonakis A, Economoua NT, Paparrigopoulos T, Bonanni E, Maestri M, Carnicelli L, et al. Sleep in frontotemporal dementia is equally or possibly more disrupted, and at an earlier stage, when compared to sleep in Alzheimer's disease. Journal of Alzheimer's Disease. 2014;38(1):85-91.

10. Bonanni E, Maestri M, Tognoni G, Fabbrini M, Nucciarone B, Manca ML, et al. Daytime sleepiness in mild and moderate Alzheimer's disease and its relationship with cognitive impairment. Journal of Sleep Research. 2005;14(3):311-7.

11. Borges CR, Piovezan RD, Poyares DR, Busatto Filho G, Studart-Neto A, Coutinho AM, et al. Subjective sleep parameters in prodromal Alzheimer's disease: a case-control study. Revista brasileira de psiquiatria (Sao Paulo, Brazil : 1999). 2020.

12. Bowen ME, Ji X, Griffioen MA. Poor Sleep Predicts Increased Pain Perception Among Adults With Mild Cognitive Impairment. Nursing research. 2021.

13. Brayet P, Petit D, Frauscher B, Gagnon JF, Gosselin N, Gagnon K, et al. Quantitative EEG of Rapid-Eye-Movement Sleep: A Marker of Amnestic Mild Cognitive Impairment. Clinical EEG and Neuroscience. 2014;47(2):134-41.

14. Bugalho P, Magrico M, Alves L, Borbinha C. Objective sleep data as predictors of cognitive decline in dementia with Lewy Bodies and Parkinson's disease. Sleep Medicine. 2021;80:273-8.

15. Bugalho P, Salavisa M, Marto JP, Borbinha C, Alves L. Polysomnographic data in Dementia with Lewy Bodies: correlation with clinical symptoms and comparison with other alpha-synucleinopathies. Sleep Medicine. 2019;55:62-8.

16. Cagnin A, Fragiacomo F, Camporese G, Turco M, Busse C, Ermani M, et al. Sleep-Wake Profile in Dementia with Lewy Bodies, Alzheimer's Disease, and Normal Aging. Journal of Alzheimer's Disease. 2017;55(4):1529-36.

17. Carlson BW, Duke J, Jones KR, Carlson JR, Craft MA, Coleman-Jackson R, et al. Sleep-Disordered Breathing and Cerebral Oxygenation During Sleep in Adults With Mild Cognitive Impairment. Research in gerontological nursing. 2018;11(6):283-92.

18. Carnicelli L, Maestri M, Di Coscio E, Tognoni G, Fabbrini M, Schirru A, et al. A longitudinal study of polysomnographic variables in patients with mild cognitive impairment converting to Alzheimer's disease. Journal of Sleep Research. 2019;28(5):e12821.

19. Cassidy-Eagle E, Siebern A, Unti L, Glassman J, O'Hara R. Neuropsychological Functioning in Older Adults with Mild Cognitive Impairment and Insomnia Randomized to CBT-I or Control Group. Clinical Gerontologist. 2017:1-9.

20. Cassidy-Eagle EL, Siebern A, Unti L, Glassman J, O'Hara R. Treating insomnia in older adults with mild cognitive impairment in residential care settings. Sleep. 2015;38:A230.

21. Cavuoto MG, Kinsella GJ, Ong B, Pike KE, Nicholas CL. Naturalistic measurement of sleep in older adults with amnestic mild cognitive impairment: Anxiety symptoms do not explain sleep disturbance. Current Alzheimer Research. 2019;16(3):233-42.

22. Chankrachang S, Senanarong V, Poungvarin N, Phanthumchinda K, Tavichachart N, Praditsuwan R, et al. The effect of Galantamine on sleep quality in Thai Alzheimer's disease patients. Journal of the Medical Association of Thailand. 2008;91(9):1343-9.

23. Chen PC, Wu D, Chen CC, Chi NF, Kang JH, Hu CJ. Rapid eye movement sleep atonia in patients with cognitive impairment. Journal of the Neurological Sciences. 2011;305(1):34-7.

24. Chen PH, Cheng FY, Cheng SJ, Shaw JS. Predicting cognitive decline in Parkinson's disease with mild cognitive impairment: A one-year observational study. Parkinson's Disease. 2020;2020:8983960.

25. Chen R, Yin Y, Zhao Z, Huang L, Huang S, Zhuang J, et al. Elevation of serum TNF-alpha levels in mild and moderate Alzheimer patients with daytime sleepiness. Journal of Neuroimmunology.

26. Chiaravalloti A, Liguori C, Nuccetelli M, Izzi F, Sancesario G, Cimini A, et al. Hypothalamic dysfunction is related to sleep impairment and CSF biomarkers in Alzheimer Disease. European Journal of Nuclear Medicine and Molecular Imaging. 2017;44(2):S601-S2.

27. Chong MS, Ayalon L, Marler M, Loredo JS, Corey-Bloom J, Palmer BW, et al. Continuous positive airway pressure reduces subjective daytime sleepiness in patients with mild to moderate Alzheimer's disease with sleep disordered breathing. Journal of the American Geriatrics Society. 2006;54(5):777‐81.

28. Chou CA, Toedebusch CD, Redrick T, Freund D, McLel, JS, et al. Comparison of single-channel EEG, actigraphy, and sleep diary in cognitively normal and mildly impaired older adults. Sleep advances : a journal of the Sleep Research Society. 2020;1(1):zpaa006.

29. Chwiszczuk L, Breitve M, Hynninen M, Gjerstad MD, Aarsl, D, et al. Higher frequency and complexity of sleep disturbances in dementia with lewy bodies as compared to Alzheimer's disease. Neurodegenerative Diseases. 2016;16(3):152-60.

30. Clough Z, Jeyapaul P, Zotova E, Holmes C. Proinflammatory cytokines and the clinical features of dementia with Lewy bodies. Alzheimer Disease and Associated Disorders.29(1):97-9.

31. Cole CS, Richards KC, Beck CC, Roberson PK, Lambert C, Furnish A, et al. Relationships among disordered sleep and cognitive and functional status in nursing home residents. Research in gerontological nursing. 2009;2(3):183‐91.

32. Cooke JR, Ancoli-Israel S, Liu L, Loredo JS, Natarajan L, Palmer BS, et al. Continuous positive airway pressure deepens sleep in patients with Alzheimer's disease and obstructive sleep apnea. Sleep medicine. 2009;10(10):1101‐6.

33. Cooke JR, Ayalon L, Palmer BW, Loredo JS, Corey-Bloom J, Natarajan L, et al. Sustained use of CPAP slows deterioration of cognition, sleep, and mood in patients with Alzheimer's disease and obstructive sleep apnea: a preliminary study. Journal of clinical sleep medicine. 2009;5(4):305‐9.

34. Cooke JR, Liu L, Natarajan L, He F, Marler M, Loredo JS, et al. The effect of sleep-disordered breathing on stages of sleep in patients with Alzheimer's disease. Behavioral sleep medicine. 2006;4(4):219-27.

35. Cooke JR, Loredo JS, Liu L, Marler M, Corey-Bloom J, Fiorentino L, et al. Acetylcholinesterase inhibitors and sleep architecture in patients with Alzheimer's disease. Drugs and Aging. 2006;23(6):503-11.

36. Cross N, Terpening Z, Duffy SL, Lewis SJG, Grunstein R, Wong K, et al. Association between Sleep Disordered Breathing and Nighttime Driving Performance in Mild Cognitive Impairment. Journal of the International Neuropsychological Society : JINS. 2017;23(6):502-10.

37. De Gennaro L, Cordone S, Truglia I, Della Marca G, Moroni F, Marzano C, et al. EEG topography of sleep and wakefulness in patients with Mild Cognitive Impairment and Alzheimer Disease: Preliminary data. Clinical Neurophysiology. 2013;124(11):e202-e3.

38. Diaz-Roman M, Pulopulos MM, Baquero M, Salvador A, Cuevas A, Ferrer I, et al. Obstructive sleep apnea and Alzheimer's disease-related cerebrospinal fluid biomarkers in mild cognitive impairment. Sleep. 2021;44(1):zsaa133.

39. Dimpfel W, Schombert L, Keplinger-Dimpfel IK, Panossian A. Effects of an adaptogenic extract on electrical activity of the brain in elderly subjects with mild cognitive impairment: a randomized, double-blind, placebo-controlled, two-armed cross-over study. Pharmaceuticals (Basel, Switzerland). 2020;13(3).

40. EA D, MA G, T K, MA B, AG G, SM A, et al. Subjective-Objective Sleep Discrepancy in Older Adults With MCI and Subsyndromal Depression. Journal of geriatric psychiatry and neurology. 2017;30(6):316-23.

41. Falck RS, Barha CK, Chan PCY, Liu-Ambrose T. Refining sleep measurement using the Motionwatch8©: how many days of monitoring do we need to get reliable estimates of sleep quality for older adults with mild cognitive impairment? Sleep Science & Practice. 2020;4(1):1-10.

42. Falck RS, Davis JC, Best JR, Chan PCY, Li LC, Wyrough AB, et al. Effect of a Multimodal Lifestyle Intervention on Sleep and Cognitive Function in Older Adults with Probable Mild Cognitive Impairment and Poor Sleep: a Randomized Clinical Trial. Journal of Alzheimer's disease. 2020.

43. Fan Y, Liang X, Han L, Shen Y, Shen B, Chen C, et al. Determinants of Quality of Life According to Cognitive Status in Parkinson's Disease. Frontiers in Aging Neuroscience. 2020;12:269.

44. Fang B, Liu H, Yang S, Xu R, Chen G. Sleep Duration, Depression, and Peptic Ulcer Recurrence in Older Patients With Mild Cognitive Impairment. Health Psychology. 2019.

45. Fang B, Yang S, Xu R, Chen G. Association between Poor Sleep Quality and Subsequent Peptic Ulcer Recurrence in Older Patients with Mild Cognitive Impairment: Examining the Role of Social Engagement. Scientific reports. 2019;9(1):2188.

46. Favaretto S, Walter U, Baracchini C, Pompanin S, Busse C, Zorzi G, et al. Accuracy of transcranial brain parenchyma sonography in the diagnosis of dementia with Lewy bodies. European Journal of Neurology. 2016.

47. Ferman TJ, Smith GE, Dickson DW, Graff-Radford NR, Lin SC, Wszolek Z, et al. Abnormal daytime sleepiness in dementia with Lewy bodies compared to Alzheimer's disease using the Multiple Sleep Latency Test. Alzheimer's Research and Therapy. 2014;6(6):76.

48. Fern, ez-Arcos A, Morenas-Rodriguez E, Santamaria J, Sanchez-Valle R, Llado A, et al. Clinical and video-polysomnographic analysis of rapid eye movement sleep behavior disorder and other sleep disturbances in dementia with Lewy bodies. Sleep. 2019;42(7):zsz086.

49. Gabelle A, Jaussent I, Hirtz C, Vialaret J, Navucet S, Grasselli C, et al. Cerebrospinal fluid levels of orexin-A and histamine, and sleep profile within the Alzheimer process. Neurobiology of Aging. 2017;53:59-66.

50. Gaeta AM, Benitez ID, Jorge C, Torres G, Dakterzada F, Minguez O, et al. Prevalence of obstructive sleep apnea in Alzheimer's disease patients. Journal of Neurology. 2020;267(4):1012-22.

51. Gauthier S, Juby A, Dalziel W, Rehel B, Schecter R. Effects of rivastigmine on common symptomatology of Alzheimers disease (EXPLORE). Current Medical Research and Opinion. 2010;26(5):1149-60.

52. Goldman JG, Ghode RA, Ouyang B, Bernard B, Goetz CG, Stebbins GT. Dissociations among daytime sleepiness, nighttime sleep, and cognitive status in Parkinson's disease. Parkinsonism and Related Disorders. 2013;19(9):806-11.

53. Gorgoni M, Lauri G, Truglia I, Cordone S, Sarasso S, Scarpelli S, et al. Parietal Fast Sleep Spindle Density Decrease in Alzheimer's Disease and Amnesic Mild Cognitive Impairment. Neural Plasticity. 2016;2016:8376108.

54. Gronewold J, Haensel R, Kleinschnitz C, Frohnhofen H, Hermann DM. Sleep-disordered breathing in hospitalized geriatric patients with mild dementia and its association with cognition, emotion and mobility. International Journal of Environmental Research and Public Health. 2019;16(5):863.

55. Guarnieri B, Adorni F, Musicco M, Appollonio I, Bonanni E, Caffarra P, et al. Prevalence of Sleep Disturbances in Mild Cognitive Impairment and Dementing Disorders: A Multicenter Italian Clinical Cross-Sectional Study on 431 Patients. Dementia and Geriatric Cognitive Disorders. 2012;33(1):50-8.

56. Guarnieri B, Maestri M, Cucchiara F, Lo Gerfo A, Schirru A, Arnaldi D, et al. Multicenter Study on Sleep and Circadian Alterations as Objective Markers of Mild Cognitive Impairment and Alzheimer's Disease Reveals Sex Differences. Journal of Alzheimer's Disease. 2020;78(4):1707-19.

57. Gunn DG, Naismith SL, Terpening Z, Lewis SJG. The relationships between poor sleep efficiency and mild cognitive impairment in Parkinson disease. Journal of Geriatric Psychiatry and Neurology. 2014;27(2):77-84.

58. Haba-Rubio J, Ouanes S, Franc Y, Marques-Vidal P, Waeber G, Vollenweider P, et al. Do diurnal cortisol levels mediate the association between sleep disturbances and cognitive impairment? Neurobiology of Aging. 2018;69:65-7.

59. Hassainia F, Petit D, Nielsen T, Gauthier S, Montplaisir J. Quantitative EEG and statistical mapping of wakefulness and REM sleep in the evaluation of mild to moderate Alzheimer's disease. European Neurology. 1997;37(4):219-24.

60. Hatfield CF, Herbert J, Van Someren EJW, Hodges JR, Hastings MH. Disrupted daily activity/rest cycles in relation to daily cortisol rhythms of home-dwelling patients with early Alzheimer's dementia. Brain. 2004;127(5):1061-74.

61. Hayes TL, Riley T, Mattek N, Pavel M, Kaye JA. Sleep habits in mild cognitive impairment. Alzheimer Disease and Associated Disorders. 2014;28(2):145-50.

62. Hita-Yanez E, Atienza M, Cantero JL. Polysomnographic and subjective sleep markers of mild cognitive impairment. Sleep. 2013;36(9):1327-34.

63. Hita-Yanez E, Atienza M, Gil-Neciga E, Cantero JL. Disturbed sleep patterns in elders with mild cognitive impairment: the role of memory decline and ApoE epsilon4 genotype. Curr Alzheimer Res.9(3):290-7.

64. Hoch CC, Reynolds CF, 3rd, Houck PR, Hall F, Berman SR, Buysse DJ, et al. Predicting mortality in mixed depression and dementia using EEG sleep variables. The Journal of neuropsychiatry and clinical neurosciences. 1989;1(4):366-71.

65. Ishikawa I, Shinno H, Ando N, Mori T, Nakamura Y. The effect of memantine on sleep architecture and psychiatric symptoms in patients with Alzheimer's disease. Acta Neuropsychiatrica. 2016;28(3):157-64.

66. Ismail Z, Herrmann N, Francis PL, Rothenburg LS, Lobaugh NJ, Leibovitch FS, et al. A SPECT study of sleep disturbances and Alzheimer's disease. Dementia and Geriatric Cognitive Disorders.27(3):254-9.

67. Jiang B, Ding C, Yao G, Yao C, Zhang Y, Ge J, et al. Polysomnographic abnormalities in patients with vascular cognitive impairment-no dementia. Sleep Medicine. 2013;14(11):1071-5.

68. Johar H, Kawan R, Emeny RT, Ladwig KH. Impaired sleep predicts cognitive decline in old people: findings from the prospective KORA age study. Sleep. 2016;39(1):217-26.

69. Kabeshita Y, Adachi H, Matsushita M, Kanemoto H, Sato S, Suzuki Y, et al. Sleep disturbances are key symptoms of very early stage Alzheimer disease with behavioral and psychological symptoms: a Japan multi-center cross-sectional study (J-BIRD). International journal of geriatric psychiatry. 2017;32(2):222‐30.

70. Kazui H, Adachi H, Kanemoto H, Yoshiyama K, Wada T, Tokumasu Nomura K, et al. Effects of donepezil on sleep disturbances in patients with dementia with Lewy bodies: An open-label study with actigraphy. Psychiatry Research. 2017;251:312-8.

71. Khou CS, Rendzia B, Watts A. Evaluation of waist-worn actigraphy monitors for the assessment of sleep in older adults with and without Alzheimer's disease. Journal of rehabilitation and assistive technologies engineering. 2018;5:2055668318777989.

72. Kim SJ, Lee JH, Lee DY, Jhoo JH, Woo JI. Neurocognitive dysfunction associated with sleep quality and sleep apnea in patients with mild cognitive impairment. American Journal of Geriatric Psychiatry. 2011;19(4):374-81.

73. Kong SDX, Hoyos CM, Phillips CL, McKinnon AC, Lin P, Duffy SL, et al. Altered heart rate variability during sleep in mild cognitive impairment. Sleep. 2020.

74. Kume Y, Kodama A, Sato K, Kurosawa S, Ishikawa T, Ishikawa S. Sleep/awake status throughout the night and circadian motor activity patterns in older nursing-home residents with or without dementia, and older community-dwelling people without dementia. International Psychogeriatrics. 2016;28(12):2001-8.

75. Lamonica HM, Hickie IB, Ip J, Irel, C, Mowszowski L, et al. Disability in older adults across the continuum of cognitive decline: Unique contributions of depression, sleep disturbance, cognitive deficits and medical burden. International Psychogeriatrics. 2019;31(11):1611-25.

76. Larsson V, Aarsl, D, Ballard C, Minthon L, Londos E. The effect of memantine on sleep behaviour in dementia with Lewy bodies and Parkinson's disease dementia. International journal of geriatric psychiatry. 2010;25(10):1030‐8.

77. Lee JH, Jung WS, Choi WH, Lim HK. Aberrant brain stem morphometry associated with sleep disturbance in drug-naive subjects with Alzheimer's disease. Neuropsychiatric Disease and Treatment. 2016;12:2089-93.

78. Leger D, Elbaz M, Dubois A, Rio S, Mezghiche H, Carita P, et al. Alzheimer's Disease Severity is Not Significantly Associated with Short Sleep: Survey by Actigraphy on 208 Mild and Moderate Alzheimer's Disease Patients. Journal of Alzheimer's Disease. 2016;55(1):321-31.

79. Leng M, Yin H, Zhang P, Jia Y, Hu M, Li G, et al. Sleep Quality and Health-Related Quality of Life in Older People With Subjective Cognitive Decline, Mild Cognitive Impairment, and Alzheimer Disease. The Journal of nervous and mental disease. 2020;208(5):387-96.

80. Levendowski DJ, Gamaldo C, St Louis EK, Ferini-Strambi L, Hamilton JM, Salat D, et al. Head position during sleep: Potential implications for patients with neurodegenerative disease. Journal of Alzheimer's Disease. 2019;67(2):631-8.

81. Li K, Luo X, Zeng Q, Jiaerken Y, Wang S, Xu X, et al. Interactions between sleep disturbances and Alzheimer's disease on brain function: a preliminary study combining the static and dynamic functional MRI. Scientific reports. 2019;9(1):19064.

82. Li Y, Wang C, Wang J, Zhou Y, Ye F, Zhang Y, et al. Mild cognitive impairment in de novo Parkinson's disease: A neuromelanin MRI study in locus coeruleus. Movement Disorders. 2019;34(6):884-92.

83. Liguori C, Chiaravalloti A, Nuccetelli M, Izzi F, Sancesario G, Cimini A, et al. Hypothalamic dysfunction is related to sleep impairment and CSF biomarkers in Alzheimer's disease. Journal of Neurology. 2017;264(11):2215-23.

84. Liguori C, Mercuri NB, Nuccetelli M, Izzi F, Bernardini S, Placidi F. Cerebrospinal Fluid Orexin Levels and Nocturnal Sleep Disruption in Alzheimer's Disease Patients Showing Neuropsychiatric Symptoms. Journal of Alzheimer's Disease. 2018;66(3):993-9.

85. Liguori C, Mercuri NB, Nuccetelli M, Izzi F, Cordella A, Bernardini S, et al. Obstructive sleep apnea may induce orexinergic system and cerebral beta-amyloid metabolism dysregulation: is it a further proof for Alzheimer's disease risk? Sleep Medicine. 2019;56:171-6.

86. Liguori C, Nuccetelli M, Izzi F, Sancesario G, Romigi A, Martorana A, et al. Rapid eye movement sleep disruption and sleep fragmentation are associated with increased orexin-A cerebrospinal-fluid levels in mild cognitive impairment due to Alzheimer's disease. Neurobiology of Aging. 2016;40:120-6.

87. Liguori C, Placidi F, Izzi F, Spanetta M, Mercuri NB, Di Pucchio A. Sleep dysregulation, memory impairment, and CSF biomarkers during different levels of neurocognitive functioning in Alzheimer's disease course. Alzheimer's Research and Therapy. 2020;12(1):5.

88. Liguori C, Romigi A, Nuccetelli M, Zannino S, Sancesario G, Martorana A, et al. Orexinergic system dysregulation, sleep impairment, and cognitive decline in Alzheimer disease. JAMA Neurology. 2014;71(12):1498-505.

89. Liguori C, Spanetta M, Izzi F, Franchini F, Nuccetelli M, Sancesario GM, et al. Sleep-Wake Cycle in Alzheimer's Disease Is Associated with Tau Pathology and Orexin Dysregulation. Journal of Alzheimer's Disease. 2020;74(2):501-8.

90. Linssen B, Bergman E, Klarenbeek P, Hoff E. Prevalence of obstructive sleep apnea at an outpatient memory clinic. Health Science Reports. 2021;4(1):e228.

91. Litvinenko IV, Krasakov IV, Tikhomirova OV. Sleep disorders in patients with dementia in Parkinson's disease. Neuroscience and Behavioral Physiology. 2013;43(2):223-8.

92. Liu G, Weinger JG, Lu ZL, Xue F, Sadeghpour S. Efficacy and safety of MMFS-01, a synapse density enhancer, for treating cognitive impairment in older adults: A randomized, double-blind, placebo-controlled trial. Journal of Alzheimer's Disease. 2015;49(4):971-90.

93. Liu S, Pan J, Lei Q, He L, Zhong B, Meng Y, et al. Spontaneous K-Complexes may be biomarkers of the progression of amnestic mild cognitive impairment. Sleep Medicine. 2020;67:99-109.

94. Liu S, Pan J, Tang K, Lei Q, He L, Meng Y, et al. Sleep spindles, K-complexes, limb movements and sleep stage proportions may be biomarkers for amnestic mild cognitive impairment and Alzheimer's disease. Sleep and Breathing. 2020;24(2):637-51.

95. Liu Y, Chen L, Huang S, Zhang C, Lv Z, Luo J, et al. Subjective Sleep Quality in Amnestic Mild Cognitive Impairment Elderly and Its Possible Relationship With Plasma Amyloid-beta. Frontiers in Neuroscience. 2020;14:611432.

96. Luboshitzky R, Shen-Orr Z, Tzischichinsky O, Maldonado M, Herer P, Lavie P. Actigraphic sleep-wake patterns and urinary 6-sulfatoxymelatonin excretion in patients with Alzheimer's disease. Chronobiology International. 2001;18(3):513-24.

97. Lucey BP, McCullough A, Landsness EC, Toedebusch CD, McLeland JS, Zaza AM, et al. Reduced non-rapid eye movement sleep is associated with tau pathology in early Alzheimer's disease. Science translational medicine. 2019;11(474):eaau6550.

98. Maestri M, Carnicelli L, Economou NT, Bonakis A, Paparrigopoulos T, Papageorgiou ST, et al. NREM sleep transient events in fronto-temporal dementia: beyond sleep stage architecture. Archives italiennes de biologie. 2015;153(2):214-24.

99. Maestri M, Carnicelli L, Tognoni G, Di Coscio E, Giorgi FS, Volpi L, et al. Non-rapid eye movement sleep instability in mild cognitive impairment: A pilot study. Sleep Medicine. 2015;16(9):1139-45.

100. Manabe Y. A Preliminary Trial in the Efficacy of Yokukansankachimpihange on REM Sleep Behavior Disorder in Dementia With Lewy Bodies. Frontiers in nutrition. 2020;7:119.

101. Manni R, Cremascoli R, Perretti C, De Icco R, Picascia M, Ghezzi C, et al. Evening melatonin timing secretion in real life conditions in patients with Alzheimer disease of mild to moderate severity. Sleep Medicine. 2019;63:122-6.

102. Markowitz JS, Gutterman EM, Lilienfeld S, Papadopoulos G. Sleep-related outcomes in persons with mild to moderate Alzheimer disease in a placebo-controlled trial of galantamine. Sleep. 2003;26(5):602‐6.

103. Matar E, Ehgoetz Martens KA, Halliday GM, Lewis SJG. Clinical features of Lewy body dementia: insights into diagnosis and pathophysiology. Journal of Neurology. 2020;267(2):380-9.

104. Matsuoka T, Imai A, Fujimoto H, Kato Y, Shibata K, Nakamura K, et al. Neural Correlates of Sleep Disturbance in Alzheimer's Disease: Role of the Precuneus in Sleep Disturbance. Journal of Alzheimer's Disease. 2018;63(3):957-64.

105. McCarter SJ, Tabatabai GM, Jong HY, ness DJ, Timm PC, Johnson KL, et al. REM sleep atonia loss distinguishes synucleinopathy in older adults with cognitive impairment. Neurology. 2020;94(1):e15-e29.

106. McKinnon A, Terpening Z, Hickie IB, Batchelor J, Grunstein R, Lewis SJG, et al. Prevalence and predictors of poor sleep quality in mild cognitive impairment. Journal of Geriatric Psychiatry and Neurology. 2014;27(3):204-11.

107. McKinnon AC, Duffy SL, Cross NE, Terpening Z, Grunstein RR, Lagopoulos J, et al. Functional Connectivity in the Default Mode Network is Reduced in Association with Nocturnal Awakening in Mild Cognitive Impairment. Journal of Alzheimer's Disease. 2017;56(4):1373-84.

108. McKinnon AC, Lagopoulos J, Terpening Z, Grunstein R, Hickie IB, Batchelor J, et al. Sleep disturbance in mild cognitive impairment Is associated with alterations in the brain's default mode network. Behavioral Neuroscience. 2016;130(3):305-15.

109. Menon RN, Radhakrishnan A, Sreedharan SE, Sarma PS, Kumari RS, Kesavadas C, et al. Do quantified sleep architecture abnormalities underlie cognitive disturbances in amnestic mild cognitive impairment? Journal of Clinical Neuroscience. 2019;67:85-92.

110. Mizuno S, Kameda A, Inagaki T, Horiguchi J. Effects of donepezil on Alzheimer's disease: The relationship between cognitive function and rapid eye movement sleep. Psychiatry and Clinical Neurosciences. 2004;58(6):660-5.

111. Moline M, Thein S, Bsharat M, Rabbee N, Kemethofer-Waliczky M, Filippov G, et al. Safety and Efficacy of Lemborexant in Patients With Irregular Sleep-Wake Rhythm Disorder and Alzheimer's Disease Dementia: Results From a Phase 2 Randomized Clinical Trial. Journal of Prevention of Alzheimer's Disease. 2021;8(1):7-18.

112. Montplaisir J, Petit D, McNamara D, Gauthier S. Comparisons between SPECT and quantitative EEG measures of cortical impairment in mild to moderate Alzheimer's disease. European neurology. 1996;36(4):197‐200.

113. Moraes W, Poyares D, Sukys-Claudino L, Guilleminault C, Tufik S. Donepezil improves obstructive sleep apnea in Alzheimer disease: a double-blind, placebo-controlled study. Chest. 2008;133(3):677‐83.

114. Moraes Wdos S, Poyares DR, Guilleminault C, Ramos LR, Bertolucci PH, Tufik S. The effect of donepezil on sleep and REM sleep EEG in patients with Alzheimer disease: a double-blind placebo-controlled study. Sleep. 2006;29(2):199‐205.

115. Moran M, Lynch CA, Walsh C, Coen R, Coakley D, Lawlor BA. Sleep disturbance in mild to moderate Alzheimer's disease. Sleep Medicine. 2005;6(4):347-52.

116. Most EIS, Aboudan S, Scheltens P, Van Someren EJW. Discrepancy between subjective and objective sleep disturbances in early-and moderate-stage alzheimer disease. American Journal of Geriatric Psychiatry. 2012;20(6):460-7.

117. Most EIS, Scheltens P, Van Someren EJW. Increased skin temperature in alzheimer's disease is associated with sleepiness. Journal of Neural Transmission. 2012;119(10):1185-94.

118. Mulin E, Zeitzer JM, Friedman L, Le Duff F, Yesavage J, Robert PH, et al. Relationship between apathy and sleep disturbance in mild and moderate Alzheimer's disease: An actigraphic study. Journal of Alzheimer's Disease. 2011;25(1):85-91.

119. Naharci MI, Ozturk A, Yasar H, Cintosun U, Kocak N, Bozoglu E, et al. Galantamine improves sleep quality in patients with dementia. Acta Neurologica Belgica. 2015;115(4):563-8.

120. Naismith SL, Hickie IB, Terpening Z, Rajaratnam SW, Hodges JR, Bolitho S, et al. Circadian misalignment and sleep disruption in mild cognitive impairment. Journal of Alzheimer's Disease. 2014;38(4):857-66.

121. Naismith SL, Pye J, Terpening Z, Lewis S, Bartlett D. "Sleep Well, Think Well" Group Program for Mild Cognitive Impairment: a Randomized Controlled Pilot Study. Behavioral sleep medicine. 2019;17(6):778‐89.

122. Naismith SL, Rogers NL, Hickie IB, MacKenzie J, Norrie LM, Lewis SJG. Sleep well, think well: Sleep-wake disturbance in mild cognitive impairment. Journal of Geriatric Psychiatry and Neurology. 2010;23(2):123-30.

123. Naismith SL, Rogers NL, Lewis SJG, Diamond K, Terpening Z, Norrie L, et al. Sleep disturbance in mild cognitive impairment: Differential effects of current and remitted depression. Acta Neuropsychiatrica. 2011;23(4):167-72.

124. O'Keeffe J, Carlson B, DeStefano L, Wenger M, Craft M, Hershey L, et al. EEG fluctuations of wake and sleep in mild cognitive impairment. Conference proceedings : Annual International Conference of the IEEE Engineering in Medicine and Biology Society IEEE Engineering in Medicine and Biology Society Annual Conference. 2017;2017:3612-5.

125. Palmer K, Mitolo M, Burgio F, Meneghello F, Venneri A. Sleep disturbance in mild cognitive impairment and association with cognitive functioning. A case-control study. Frontiers in Aging Neuroscience. 2018;10:360.

126. Pao WC, Boeve BF, Ferman TJ, Lin SC, Smith GE, Knopman DS, et al. Polysomnographic findings in dementia with lewy bodies. Neurologist. 2013;19(1):1-6.

127. Park JE, Lee YJ, Byun MS, Yi D, Lee JH, Jeon SY, et al. Differential associations of age and Alzheimer's disease with sleep and rest-activity rhythms across the adult lifespan. Neurobiology of Aging. 2021;101:141-9.

128. Park M, Hood MM, Shah RC, Fogg LF, Wyatt JK. Sleepiness, parkinsonian features and sustained attention in mild Alzheimer's disease. Age and Ageing. 2012;41(6):765-70.

129. Park M, Shah RC, Fogg LF, Wyatt JK. Daytime sleepiness in mild Alzheimer's disease with and without parkinsonian features. Sleep Medicine. 2011;12(4):397-402.

130. Pat-Horenczyk R, Klauber MR, Shochat T, Ancoli-Israel S. Hourly profiles of sleep and wakefulness in severely versus mild- moderately demented nursing home patients. Aging Clinical and Experimental Research. 1998;10(4):308-15.

131. Petit D, Montplaisir J, Lorrain D, Gauthier S. THA does not affect sleep or EEG spectral power in Alzheimer's disease. Biological Psychiatry.33(10):753-4.

132. Petit D, Montplaisir J, Lorrain D, Gauthier S. Spectral analysis of the rapid eye movement sleep electroencephalogram in right and left temporal regions: A biological marker of Alzheimer's disease. Annals of Neurology. 1992;32(2):172-6.

133. Pini L, Wennberg A, Mitolo M, Meneghello F, Burgio F, Semenza C, et al. Quality of sleep predicts increased frontoparietal network connectivity in patients with mild cognitive impairment. Neurobiology of Aging. 2020;95:205-13.

134. Pinol-Ripoll G, Targa A, Benitez I, Dakterzada F, Lopez R, Barbe F. Decrease in sleep depth is associated with higher cerebrospinal fluid neurofilament light levels in Alzheimer's disease patients. Journal of Sleep Research. 2020;29.

135. Prinz PN, Larsen LH, Moe KE, Vitiello MV. EEG markers of early Alzheimer's disease in computer selected tonic REM sleep. Electroencephalography and Clinical Neurophysiology. 1992;83(1):36-43.

136. Prinz PN, Vitaliano PP, Vitiello MV, Bokan J, Raskind M, Peskind E, et al. Sleep, EEG and mental function changes in senile dementia of the Alzheimer's type. Neurobiology of Aging. 1982;3(4):361-70.

137. Ratti PL, Terzaghi M, Minafra B, Repetto A, Pasotti C, Zangaglia R, et al. REM and NREM sleep enactment behaviors in Parkinson's disease, Parkinson's disease dementia, and dementia with Lewy bodies. Sleep Medicine. 2012;13(7):926-32.

138. Rauchs G, Piolino P, Bertran F, de La Sayette V, Viader F, Eustache F, et al. Retrieval of recent autobiographical memories is associated with slow-wave sleep in early AD. Frontiers in Behavioral Neuroscience. 2013.

139. Rawtaer I, Mahendran R, Kua EH, Tan HP, Tan HX, Lee TS, et al. Early detection of mild cognitive impairment with in-home sensors to monitor behavior patterns in community-dwelling senior citizens in Singapore: Cross-sectional feasibility study. Journal of Medical Internet Research. 2020;22(5):e16854.

140. Reda F, Gorgoni M, Lauri G, Truglia I, Cordone S, Scarpelli S, et al. In search of sleep biomarkers of Alzheimer's disease: K-complexes do not discriminate between patients with mild cognitive impairment and healthy controls. Brain Sciences. 2017;7(5):51.

141. Rongve A, Boeve BF, Aarsl, D. Frequency and correlates of caregiver-reported sleep disturbances in a sample of persons with early dementia. Journal of the American Geriatrics Society. 2010;58(3):480-6.

142. Rosales-Lagarde A, ra, Rodriguez-Torres EE, Itza-Ortiz BA, Miramontes P, Vazquez-Tagle G, et al. The Color of Noise and Weak Stationarity at the NREM to REM Sleep Transition in Mild Cognitive Impaired Subjects. Frontiers in psychology. 2018;9:1205.

143. Rose KM, Beck C, Tsai PF, Liem PH, Davila DG, Kleban M, et al. Sleep disturbances and nocturnal agitation behaviors in older adults with dementia. Sleep. 2011;34(6):779-86.

144. Rozzini L, Conti MZ, Riva M, Ceraso A, Caratozzolo S, Zanetti M, et al. Non-amnestic mild cognitive impairment and sleep complaints: a bidirectional relationship? Aging Clinical and Experimental Research. 2018;30(6):661-8.

145. Sanchez-Espinosa MP, Atienza M, Cantero JL. Sleep deficits in mild cognitive impairment are related to increased levels of plasma amyloid-beta and cortical thinning. NeuroImage. 2014;98:395-404.

146. Sanchez-Espinosa MP, Atienza M, Cantero JL. Sleep mediates the association between homocysteine and oxidative status in mild cognitive impairment. Scientific Reports. 2017;7.

147. Saricaoglu M, Yilmaz NH, Ozer FF, Hanoglu L. The correlation of non-motor symptoms and sleep on balance in Parkinson's disease patients with normal cognition and mild cognitive impairment. Irish Journal of Medical Science. 2021.

148. Scharre DW, Chang SI, Nagaraja HN, Park A, Adeli A, Agrawal P, et al. Paired Studies Comparing Clinical Profiles of Lewy Body Dementia with Alzheimer's and Parkinson's Diseases. Journal of Alzheimer's Disease. 2016;54(3):995-1004.

149. Scherder E, Knol D, van Someren E, Deijen JB, Binnekade R, Tilders F, et al. Effects of low-frequency cranial electrostimulation on the rest-activity rhythm and salivary cortisol in Alzheimer's disease. Neurorehabilitation and neural repair. 2003;17(2):101‐8.

150. Scullin MK, Trotti LM, Wilson AG, Greer SA, Bliwise DL. Nocturnal sleep enhances working memory training in Parkinson's disease but not Lewy body dementia. Brain. 2012;135(9):2789-97.

151. Seidel S, Dal-Bianco P, Pablik E, Muller N, Schadenhofer C, Lamm C, et al. Depressive symptoms are the main predictor for subjective sleep quality in patients with mild cognitive impairment - A controlled study. PLoS ONE. 2015;10(6):e0128139.

152. Sobreira EST, Sobreira-Neto MA, Pena-Pereira MA, Chagas MHN, Fern, es RMF, et al. Global cognitive performance is associated with sleep efficiency measured by polysomnography in patients with Parkinson's disease. Psychiatry and Clinical Neurosciences. 2019;73(5):248-53.

153. Song D, Yu DSF. Effects of a moderate-intensity aerobic exercise programme on the cognitive function and quality of life of community-dwelling elderly people with mild cognitive impairment: a randomised controlled trial. International journal of nursing studies. 2019;93:97‐105.

154. Song D, Yu DSF, Li PWC, He G, Sun Q. Correlates of health-related quality of life among Chinese older adults with mild cognitive impairment. Clinical Interventions in Aging. 2019;14:2205-12.

155. Song D, Yu DSF, Sun Q, He G. Correlates of sleep disturbance among older adults with mild cognitive impairment: A cross-sectional study. International Journal of Environmental Research and Public Health. 2020;17(13):1-12.

156. Song Y, McCurry SM, Lee D, Josephson KR, McGowan SK, Fung CH, et al. Development of a dyadic sleep intervention for Alzheimer's disease patients and their caregivers. Disability and rehabilitation. 2019:1-11.

157. Soysal P, Tan SG. The prevalence and co-incidence of geriatric syndromes in older patients with early-stage Alzheimer's disease and dementia with Lewy bodies. Aging Clinical and Experimental Research. 2021.

158. Spira AP, Yager C, Br, t J, Smith GS, Zhou Y, et al. Objectively Measured Sleep and beta-amyloid Burden in Older Adults: A Pilot Study. SAGE open medicine. 2014;2.

159. Stanzani-Maserati M, Gallassi R, Cal, ra-Buonaura G, Aless, ria M, et al. Cognitive and sleep features of multiple system atrophy: Review and prospective study. European Neurology. 2014;72(5):349-59.

160. Sun Q, Luo L, Ren H, Wei C, Xing M, Cheng Y, et al. Semantic clustering and sleep in patients with amnestic mild cognitive impairment or with vascular cognitive impairment-no dementia. International Psychogeriatrics. 2016;28(9):1493-502.

161. Tadokoro K, Ohta Y, Hishikawa N, Nomura E, Wakutani Y, Takao Y, et al. Discrepancy of subjective and objective sleep problems in Alzheimer's disease and mild cognitive impairment detected by a home-based sleep analysis. Journal of Clinical Neuroscience. 2020;74:76-80.

162. Taheri M, Ir, oust K, Modabberi S. An acute bout of dynamic sitting exercises improves stroop performance and quality of sleep in older adults with cognitive impairment. International archives of health sciences. 2019;6(4):126‐30.

163. Taillard J, Sagaspe P, Berthomier C, Br, ewinder M, Amieva H, et al. Non-REM sleep characteristics predict early cognitive impairment in an aging population. Frontiers in Neurology. 2019;10:197.

164. Tajiri Y, Wada-Isoe K, Tanaka K, Adachi T, Hanajima R, Nakashima K. A single-institution study on predictors of short-term progression from mild cognitive impairment in Parkinson's disease to Parkinson's disease with dementia. Yonago Acta Medica. 2020;63(1):28-33.

165. Targa A, Dakterzada F, Benitez I, Lopez R, Pujol M, Dalmases M, et al. Decrease in sleep depth is associated with higher cerebrospinal fluid neurofilament light levels in patients with Alzheimer's disease. Sleep. 2021;44(2).

166. Terpening Z, Lewis SJG, Yee BJ, Grunstein RR, Hickie IB, Naismith SL. Association between sleep-disordered breathing and neuropsychological performance in older adults with mild cognitive impairment. Journal of Alzheimer's Disease. 2015;46(1):157-65.

167. Terzaghi M, Arnaldi D, Rizzetti MC, Minafra B, Cremascoli R, Rustioni V, et al. Analysis of video-polysomnographic sleep findings in dementia with Lewy bodies. Movement Disorders. 2013;28(10):1416-23.

168. Terzaghi M, Minafra B, Zangaglia R, Picascia M, Pozzi N, Cremascoli R, et al. NREM sleep arousal-related disorders reflect cognitive impairment in Parkinson's disease. Sleep Medicine. 2020;75:491-6.

169. Thomas NWD, Beattie Z, Marcoe J, Wright K, Sharma N, Mattek N, et al. An Ecologically Valid, Longitudinal, and Unbiased Assessment of Treatment Efficacy in Alzheimer Disease (the EVALUATE-AD Trial): Proof-of-Concept Study. JMIR research protocols. 2020;9(5):e17603.

170. Vitiello MV, Prinz PN, Williams DE, Frommlet MS, Ries RK. Sleep disturbances in patients with mild-stage Alzheimer's disease. Journals of Gerontology. 1990;45(4):M131-M8.

171. Wade AG, Farmer M, Harari G, Fund N, Laudon M, Nir T, et al. Add-on prolonged-release melatonin for cognitive function and sleep in mild to moderate Alzheimer's disease: a 6-month, randomized, placebo-controlled, multicenter trial. Clinical interventions in aging. 2014;9:947â61.

172. Wams EJ, Wilcock GK, Foster RG, Wulff K. Sleep-wake patterns and cognition of older adults with amnestic mild cognitive impairment (aMCI): A comparison with cognitively healthy adults and moderate Alzheimer's disease patients. Current Alzheimer Research. 2017;14(10):1030-41.

173. Wang JJ, Shih YH, Pai MC, Huang YC. Sundown Syndrome, Sleep Quality, and Walking Among Community-Dwelling People With Alzheimer Disease. Journal of the American Medical Directors Association. 2017;18(5):396-401.

174. Wang L, Wu B, Tao H, Chai N, Zhao X, Zhen X, et al. Effects and mediating mechanisms of a structured limbs-exercise program on general cognitive function in older adults with mild cognitive impairment: a randomized controlled trial. International journal of nursing studies. 2020;110:103706‐.

175. Wang Y, Cheng C, Gooneratne N, Carter P, Richards K. One year of CPAP adherence improves cognition in older adults with mild apnea and mild cognitive impairment. Sleep. 2019;42:A281.

176. Weissova K, Bartos A, Sladek M, Novakova M, Sumova A. Moderate changes in the circadian system of Alzheimer's disease patients detected in their home environment. PLoS ONE. 2016;11(1):e0146200.

177. Westerberg CE, er BA, Florczak SM, Weintraub S, Mesulam MM, Zee PC, et al. Concurrent impairments in sleep and memory in amnestic mild cognitive impairment. Journal of the International Neuropsychological Society. 2012;18(3):490-500.

178. Westerberg CE, Lundgren EM, Florczak SM, Mesulam MM, Weintraub S, Zee PC, et al. Sleep influences the severity of memory disruption in amnestic mild cognitive impairment: Results from Sleep self-assessment and continuous activity monitoring. Alzheimer Disease and Associated Disorders. 2010;24(4):325-33.

179. Wilson G, Terpening Z, Wong K, Grunstein R, Norrie L, Lewis SJG, et al. Screening for sleep apnoea in mild cognitive impairment: The utility of the multivariable apnoea prediction index. Sleep Disorders. 2014:945287.

180. Wood KH, Memon AA, Memon RA, Joop A, Pilkington J, Catiul C, et al. Slow Wave Sleep and EEG Delta Spectral Power are Associated with Cognitive Function in Parkinson's Disease. Journal of Parkinson's disease. 2020.

181. Xie LQ, Yang BX, Liao YH, Gao GX, Jiang N, Zhou J, et al. Sleep Disturbance in Older Adults With or Without Mild Cognitive Impairment and Its Associated Factors Residing in Rural Area, China. Journal of Geriatric Psychiatry and Neurology. 2020.

182. Yesavage JA, Taylor JL, Kraemer H, Noda A, Friedman L, Tinklenberg JR. Sleep/wake cycle disturbance in Alzheimer's disease: How much is due to an inherent trait? International Psychogeriatrics. 2002;14(1):73-81.

183. Yohannes AM, M NE, Holbrook JT, Sugar EA, Henderson R, Baker AM, et al. Association of mild cognitive impairment and characteristic of COPD and overall health status in a cohort study. Expert Review of Respiratory Medicine. 2021;15(1):153-9.

184. You JC, Jones E, Cross DE, Lyon AC, Kang H, Newberg AB, et al. Association of β-Amyloid Burden With Sleep Dysfunction and Cognitive Impairment in Elderly Individuals With Cognitive Disorders. JAMA Network Open. 2019;2(10):e1913383-e.

185. Yu J, Mahendran R, Rawtaer I, Kua EH, Feng L. Poor sleep quality is observed in mild cognitive impairment and is largely unrelated to depression and anxiety. Aging and Mental Health. 2016:1-6.

186. Yu JM, Tseng IJ, Yuan RY, Sheu JJ, Liu HC, Hu CJ. Low sleep efficiency in patients with cognitive impairment. Acta Neurologica Taiwanica. 2009;18(2):91-7.

187. Yuen K, Rashidi-Ranjbar N, Verhoeff NPLG, Kumar S, Gallagher D, Flint AJ, et al. Association between Sleep Disturbances and Medial Temporal Lobe Volume in Older Adults with Mild Cognitive Impairment Free of Lifetime History of Depression. Journal of Alzheimer's Disease. 2019;69(2):413-21.

188. Zhang L, Li T, Lei Y, Cheng G, Liu B, Yu Y, et al. Association between sleep structure and amnesic mild cognitive impairment in patients with insomnia disorder: A case-control study. Journal of Clinical Sleep Medicine. 2021;17(1):37-43.

Section 4 – Full Table of Validated Questionnaires Within Included Articles

| Validated Questionnaire | Abbreviation |
| --- | --- |
| Pittsburgh Sleep Quality Index | PSQI |
| Clinical Global Impression of Change | CGI-C |
| Epworth Sleepiness Scale | ESS |
| Neuropsychiatry Inventory (Sleep Disturbance) | NPI |
| REM Sleep Behavioural Disorder Screening Questionnaire | RSBD-SQ |
| Mayo Sleep Questionnaire | MSQ |
| Consensus Sleep Diary | - |
| Insomnia Severity Index | ISI |
| Athens Insomnia Index | - |
| BEHAVE-AD Questionnaire | BEHAVE-AD |
| Berlin Questionnaire | - |
| Jupiter Medical Centre Sleep Questionnaire | - |
| Neurodegenerative Disease Sleep Questionnaire | NDSQ |
| Non-Motor Symptoms Questionnaire | NMSQ |
| Panic Disorder Severity Scale | PDSS |
| Schlaffragebogen SF-B | SF-B/R |
| Sleep Continuity in Alzheimer‘s Disease | SCAD |
| Sleep Disturbance Symptom Questionnaire | SDSQ |
| Uppsala Sleep Inventory | USI |
| Visual Analog Scales | - |
